# Size, Conformation, and Local Domains of Single-Chain Nanoparticles with Intrachain Covalent, Dipolar, and Electrostatic Interactions: Toward Artificial Intrinsically Disordered Proteins

**DOI:** 10.1021/acs.macromol.5c01317

**Published:** 2025-07-24

**Authors:** Mikel Iguaran, Sara Gutierrez-Lkourt, Ester Verde-Sesto, Armando Maestro, José A. Pomposo

**Affiliations:** † 202635 Materials Physics Center (CFM-MPC), CSIC-UPV/EHU, Paseo Manuel de Lardizabal 5, 20018 Donostia, Spain; ‡ IKERBASQUE − Basque Foundation for Science, Plaza Euskadi 5, 48009 Bilbao, Spain; § Department of Polymers and Advanced Materials: Physics, Chemistry and Technology, University of the Basque Country (UPV/EHU), Paseo Manuel de Lardizabal 3, 20800 Donostia, Spain

## Abstract

Herein, we develop the hierarchical scaling model of single-chain polymer nanoparticles (SCNPs) with intrachain covalent bonds (i.e., intramolecular cross-links) and dipolar and electrostatic interactions, as simplified analogues of intrinsically disordered proteins (IDPs). By combining the standard elastic single-chain nanoparticle model with the mean-field dipole theory and polyelectrolyte scaling laws, we uncover new insights into the size and conformation of SCNPs with multiple interactions at different scales in a good solvent, at high dilution, and in semidiluted solutions, the latter being the most relevant conditions to compare with experimental data. The model takes into account key parameters such as the type of monomers involved, the composition and length of the precursor chain, the elasticity characteristics of the SCNPs upon intrachain cross-linking, the interaction strengths, and both the SCNPs and (monovalent) salt concentrations in solution. Our findings reveal distinct scaling behaviors depending on the balance between attractive dipolar and repulsive excluded volume forces. Notably, we demonstrate that electrostatic interactions above a threshold of charged monomers induce extended SCNP conformations at high dilution (below the chain overlap concentration, c*), while salt addition screens these effects, resulting in more collapsed configurations. In semidiluted salt-free solutions (*c* > c*), these SCNPs adopt a random walk conformation at high concentration. We provide useful expressions to estimate the size and number of local compact domains in these complex SCNPs, which can be exploited to immobilize catalysts, luminophores, or drugs. At the scaling level of accuracy, this theoretical approach offers a comprehensive examination of how multiple short- and long-range forces affect SCNP configuration from a local to a large scale, enabling the rational design of artificial IDPs for applications in nanomedicine, antifouling coatings, and stimuli-responsive materials. As a practical example, we provide useful design principles for the construction of expanded or, conversely, compact artificial IDPs based on SCNPs with covalent, dipolar, and electrostatic interactions.

## Graphical abstract

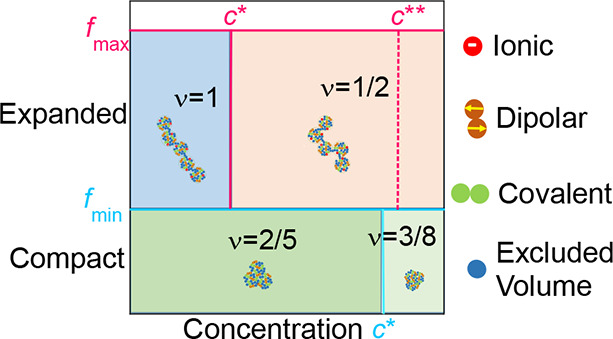

## 1 Introduction

The folding/collapse of individual synthetic polymer chains into single-chain polymer nanoparticles (SCNPs)^1^ via intrachain interactions—mimicking the folding-promoted functionality of some biopolymers—has paved the way to the development of innovative catalysts,^2–6^ sensors,^7–11^ and drug delivery vehicles.^12–16^ The internal confinement effect upon SCNP formation^17–19^ can be exploited to immobilize catalysts, luminophores, or drugs within the resulting local pockets of the ultrafine SCNPs.^20–29^ Interestingly, entrapment within such specific nanocavities can be adjusted from permanent to merely transient.^30,31^ How to design, construct, and characterize well-defined, functional nanocompartments within SCNPs is one of the still open questions in the field.^32^ A reliable theoretical model could be especially useful to assist in the former.

In nature, the folding of proteins, DNA, or RNA is guided by a variety of interactions,^33^ including hydrophobic forces, hydrogen bonding, dipole–dipole forces, π–π stacking, disulfide reversible bonds, etc. Conversely, the folding/collapse of synthetic SCNPs often involves the use of only one or two different kinds of interactions, due to the synthetic difficulties of incorporating multiple orthogonal reactive functional groups/interactions to promote the folding/collapse of the precursor of the SCNPs.^34^ Molecular dynamics (MD) simulations have been employed to investigate the effect of multiple (orthogonal) covalent interactions on the size and conformation of conventional SCNPs.^35,36^ Several attempts have been carried out to provide theoretical models to gain insight into the main factors affecting the folding/collapse of individual synthetic polymer chains into SCNPs^37–40^ and even to understand the locally compact domains of SCNPs.^41^ In general, the models^37–40^ assume that the precursor chain is neutral, nonamphiphilic, and completely flexible. In particular, the elastic single-chain nanoparticle (ESN) model^40^ allows one to understand the effect of precursor size, intrachain cross-linking degree, and solvent quality on the size and conformation of covalent-bonded SCNPs in solution and when deposited on surfaces of different surface energy values. The standard ESN model has been employed with success to determine the size of elastic SCNPs in nanopores,^42^ as well as anchored to a flat surface in the form of a dense brush of SCNPs.^43^ Moreover, the size and conformation of superstructures of increasing complexity composed by stars, combs, and bottlebrushes of SCNPs have been investigated within the framework of the ESN model.^44^ More recently, the ESN model has been combined with the classical scaling theory of polyelectrolyte (PE) solutions^45,46^ to understand why SCNPs from a weak PE can be synthesized at a large scale in a concentrated solution without intermolecular aggregation issues.^47^ Importantly, the extended model provides a simple and convincing explanation: the relevant length scale in PE-SCNPs is the local electrostatic blob size, rather than the total chain size in the case of SCNPs prepared from neutral chains.^47^

Motivated by the success of the ESN model incorporating both short-range interactions (excluded volume) and long-range (electrostatic) forces, we investigated the size and conformation of SCNPs with intrachain covalent bonds (i.e., intramolecular cross-links) and dipolar and electrostatic interactions in solution at different scales. As a source of dipolar interaction, we consider the presence in the SCNPs of zwitterionic monomers involved in dipole–dipole attraction forces.^48^ Synthetic polyzwitterions, as a special type of polyampholytes bearing side groups carrying both positive and negative charges on the same monomeric unit, have been known since 1957.^49^ Some polyzwitterions are thermoresponsive in solution and show screening of attractive dipolar interactions upon salt addition (i.e., anti-polyelectrolyte behavior).^50^ Although zwitterionic macromolecules show great potential as vehicles to aid in drug and gene delivery, antifreeze and antifouling coatings, hydrogel materials, and so on,^51^ their use as precursors of SCNPs is still rather limited.^8,52^ Indeed, the large number of parameters on which the size and conformation of SCNPs with intrachain covalent, dipolar, and long-range electrostatic interactions are likely to depend precludes the use of extensive MD simulations to guide experimental design due to exacerbated computational costs, so theoretical approaches are certainly welcome. To name a few, the size and conformation of these SCNPs are expected to depend on the fraction of reactive, dipolar, and electrostatic monomers in the precursor, the total number of monomers and the monomer length, the strength of the dipolar interactions, the elasticity constant of the SCNPs, the Bjerrum length, the concentration of added salt, etc. Therefore, theoretical models become essential to guide and facilitate the rational design of functional SCNPs with intrachain covalent, dipolar, and electrostatic interactions, as simplified analogues of intrinsically disordered proteins (IDPs). The conformations of linear polyampholyte (PA) and polyzwitterion chains as simplified versions of IDPs have been investigated through different theoretical approaches, from classical Flory-like free energy expressions^53^ to simple scaling^54,55^ and more elaborated variational approaches.^56–59^ Interestingly, the complete scaling diagram of conformational regimes for a PA with random charge statistics in a salt-added solution, as a simplified model of polyampholytic IDPs, has been recently reported by Rumyantsev et al.^60^ Relevant theoretical frameworks beyond the scaling level of accuracy for systems involving electrostatic interactions can be found in refs 61–67 .

Herein, we develop the hierarchical scaling model of SCNPs with intrachain covalent, dipolar, and electrostatic interactions in a good solvent at high dilution (below the chain overlap concentration, c*) both with and without added salt. The model, in addition to the total size and conformation of these SCNPs, provides useful expressions to estimate the local domain size and number of nanocompartments in these complex SCNPs. As is usual when working with scaling models, we discard microscopic details (e.g., specific monomer sequences, chain stiffness effects, dipole directional restrictions, counterion sizes, multivalent counterions) and nonequilibrium effects—requiring refined theories^67–74^ —to focus on the physical effects most important for understanding the general qualitative behavior at thermodynamic equilibrium. Additionally, we investigate the size and configuration of these SCNPs in semidiluted solutions (*c* > c*), which are the conditions more relevant for comparison with IDPs, as the natural counterparts of ionic SCNPs.^17,30–32^ Very recently, a detailed analysis of the intrinsically disordered human proteome has revealed how chain compaction in IDPs and regions (collectively, IDRs) relates to their cellular function and localization.^75^ Significant enrichment of compact IDRs was found in proteins that bind chromatin and DNA *cis*-regulatory sequences. On the contrary, expanded IDRs were associated with proteins that bind GTP or have G-protein-coupled activity. Hence, chromatin and nuclear bodies are cellular components enriched in compact IDRs, whereas mitochondria and endosomes are enriched in expanded IDRs. We will use the model to provide design principles toward artificial IDPs based on SCNPs with covalent, dipolar, and electrostatic interactions having these two opposite configurations: extended vs compact, both in a good solvent (water).

The remainder of the paper is organized as follows. Section 2 describes the theoretical approach. In Section 2.1, we obtain expressions for the size and conformation of a precursor chain of nonionic SCNPs with intrachain covalent and dipolar interactions in a very good solvent at high dilution as a function of the balance of attractive dipolar interactions and repulsive excluded volume effects. In Section 2.2, we investigate the size and configuration of nonionic SCNPs with intrachain covalent and dipolar interactions in different conditions at different length scales. Sections 2.3 and 2.4 are equivalent to Sections 2.1 and 2.2 but for the precursor of ionic SCNPs involving intrachain covalent, dipolar, and electrostatic interactions and the ionic SCNPs thereof, respectively. Finally, Section 3 is devoted to discussion, and Section 4 is devoted to conclusions.

## 2 Theoretical Approach

### 2.1 Size and Conformation of a Linear Precursor of Single-Chain Nanoparticles with Dipolar Interactions in a Diluted Solution

In this section, we consider a flexible polymer chain containing neutral monomers (inert and reactive) as well as zwitterionic (dipolar) monomers as the SCNP precursor. We denote as *x* and *s* the fractions of reactive and zwitterionic monomers in the precursor chain, respectively. Accordingly, the number of inert monomers is *N*–*xN*–*sN* = *N*(1–*x*–*s*), where *N* is the total number of monomers in a single precursor chain. For simplicity, we assume that all of the monomers have the same size, *b*, and are randomly distributed along the chain (see Figure 1). We will specifically look at how the chain behaves in a good solvent that is highly diluted both with and without additional salt.

**Figure 1:**
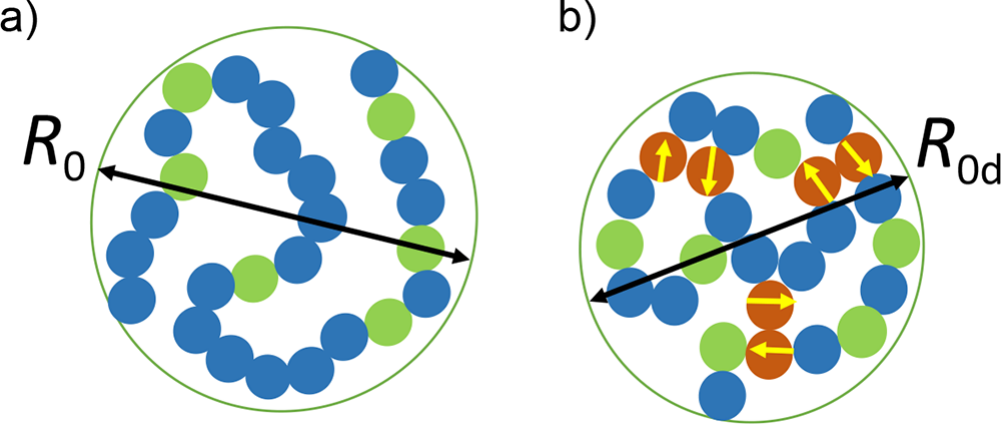
(a) Cartoon to illustrate the size (*R*_0_) of a flexible precursor chain in diluted solution containing only *xN* reactive monomers (green color) and (1–*x*)*N* inert monomers (blue color), where *N* is the total number of monomers in the chain. (b) Size (*R*_0d_) of an equivalent precursor chain with *xN* reactive monomers (green color), *sN* zwitterionic monomers (brown color) that are involved in pairwise dipole–dipole interactions between them to give quadrupoles (yellow arrows), and *N*(1–*x*–*z*) inert monomers (blue color).

#### 2.1.1 Precursor in a Good Solvent at High Dilution (No Added Salt): Size and Conformation

The size and conformation of a polymer chain with dipolar interactions have been investigated through different theoretical approaches, from simple Flory-like free energy expressions^53^ to scaling therory^54,55^ and more sophisticated variational approaches.^56–59^ Here, we consider the specific case of a statistical (random) copolymer comprising neutral and zwitterionic monomers as precursors of the SCNPs. The expression of the free energy (*F*) of a linear chain containing zwitterionic monomers in a very good solvent at high dilution can be written, according to the mean-field dipole (MFD) theory of polyzwitterion chains developed by Muthukumar,^76^ as

$$\beta F={\beta F}_{1}+{\beta F}_{2}+{\beta F}_{3}+{\beta F}_{4}$$ 1

where β ≡ (*k*_B_ *T*)^−1^, *k*_B_ is the Boltzmann constant, and *T* is the absolute temperature. *F*_1_ corresponds to the contribution to the free energy coming from the conventional entropic elasticity of the chain:^77^

$${\beta F}_{1}\approx \frac{R^{2}}{b^{2}N}$$ 2

where *R* is the size (end-to-end distance) of the precursor chain. We introduce the symbol “≈” to indicate that for simplicity we consider the prefactor on the r.h.s of eq 2 to be around unity. *F*_2_ and *F*_3_ account for binary and ternary excluded volume interactions that prevent the monomers from overlapping:^77^

$$\beta F_{2}=v_{\mathrm{ev}}\frac{N^{2}}{R^{3}}$$ 3

$${\beta F}_{3}=w\frac{N^{3}}{R^{6}}$$ 4

with *v*_ev_ = *b* ^3^ and *w* = *b* ^6^ in the limiting case of a very good (athermal) solvent. *F*_4_ corresponds to the contribution from the dipole–dipole interactions between *sN* zwitterionic monomers in the precursor chain (see Figure 1b):

$$\beta F_{4}=v_{d}\frac{(sN{)}^{2}}{R^{3}}$$ 5

Notice that dipole–dipole interactions are attractive, *v*_d_ < 0, in contrast to repulsive excluded volume interactions in a very good solvent, *v*_ev_ > 0. Analytical expressions for *v*_d_ have been derived by Muthukumar^76^ for the case of randomly oriented dipoles (high temperature) and quenched quadrupoles (low temperature) as a function of the Bjerrum length $l_{B}=\frac{e^{2}}{4\pi \varepsilon k_{B}T}$ (where *e* is the elementary charge and ε is the dielectric constant of the solvent), the dipole length *p*_0_, the separation distance between dipoles *r*_0_, and the inverse Debye length κ, which is proportional to the square root of the concentration of added salt in the solution *c*_s_. Since in this case we assume no salt is added to the solution, we denote the interaction strength as *v*_d0_.

Accordingly, eq 1 becomes

$$\beta F\approx \frac{R^{2}}{b^{2}N}+v_{\mathrm{eff}}\frac{N^{2}}{R^{3}}+b^{6}\frac{N^{3}}{R^{6}}$$ 6

where *v*_eff_ ≡ *v*_ev_ + *s* ^2^ *v*_d0_ combines repulsive and attractive interactions in an effective second virial coefficient.^78^ Minimization of eq 6 with respect to *R* provides an expression to determine the value of the equilibrium size of the precursor chain (*R*_0d_).

$$\frac{2R_{0d}}{b^{2}N}-3(b^{3}+s^{2}v_{d0})\frac{N^{2}}{R_{0d}^{4}}-6b^{6}\frac{N^{3}}{R_{0d}^{7}}=0$$ 7

Importantly, three limiting scaling laws can be derived from eq 7 depending on the balance between attractive dipolar forces (*s* ^2^ *v*_d0_ < 0) and repulsive excluded volume interactions (*v*_ev_ = *b* ^3^ > 0).

- If the attractive dipolar interactions are much weaker than the repulsive interactions (i.e., |*s* ^2^ *v*_d0_| ≪ *b* ^3^), we have

$$R_{0d}\approx b{\left(1+\frac{s^{2}v_{d0}}{b^{3}}\right)}^{1/5}N^{3/5}$$ 8

eq 8 reduces to the classical expression for a self-avoiding chain (*R*_0_ ≈ *bN* ^3/5^) in the case of a precursor chain containing no zwitterionic monomers (*s* = 0).^76^ Remarkably, eq 8 can also be obtained through a classical scaling approach just by replacing *v*_ev_ by *v*_eff_ in the derivation of the thermal blob size $\left(\xi_{T}\approx \frac{b^{4}}{\left|v_{\mathrm{eff}}\right|}\right)$ .^77^ The presence of dipolar interactions does not modify the self-avoiding walk (SAW) conformation of the chain^77^ (*R*_0d_ ∝ *N* ^3/5^) but does reduce its size. The relative size reduction due to the presence of dipolar interactions is

$$\frac{R_{0d}}{R_{0}}\approx {\left(1+\frac{s^{2}v_{d0}}{b^{3}}\right)}^{1/5}$$ 9

- If the attractive forces balance with the repulsive ones (i.e., |*s* ^2^ *v*_d0_| = *b* ^3^), the precursor chain adopts the conformation of a Gaussian chain^77^ (random walk, RW) with $R_{0d}\approx {bN}^{1/2}$ even being in a very good solvent (*v*_ev_ ≠ 0).
- If the attractive dipole–dipole forces become much stronger (i.e., |*s* ^2^ *v*_d0_| ≫ *b* ^3^), the conformation of the precursor chain becomes globular^77^ (*R*_0d_ ∝ *N* ^1/3^), with the size given by

$$R_{0d}\approx b{\left(\left|1+\frac{s^{2}v_{d0}}{b^{3}}\right|\right)}^{-1/3}N^{1/3}$$ 10

It is worth mentioning that similar limiting expressions for the case of alternating PAs can be obtained by means of the random phase approximation as pioneered by Wittmer, Johner, and Joanny^78^ as well as through the recent scaling theory of neutral sequence-specific PA by Rumyantsev, Jackson, Johner, and de Pablo.^55^

Concerning the case |*s* ^2^ *v*_d0_| ≫ *b* ^3^, the solvent becomes a bad solvent for the precursor chain since *v*_eff_ < 0. In real experiments, multichain aggregation phenomena and even macroscopic precipitation of the polymer out of the solution are expected. Interestingly, this fact allows one to estimate the value of *v*_d0_ from the fraction of zwitterionic monomers in the precursor that makes the chain insoluble in the solvent (*s*_max_). By assuming *b* ^3^ + *s*_max_^2^ *v*_d0_ = 0 at *s* ∼ *s*_max_, we obtain

$$v_{d0}=-\frac{b^{3}}{s_{\max}^{2}}$$ 11

For example, random copolymers of methacrylate and phosphorylcholine-based zwitterionic monomers typically become insoluble in chloroform when *s* > 0.24–0.26 (i.e.*, s*_max_ ∼ 0.25).^79^ Therefore, we can estimate the strength of the dipolar interactions at the point where the precursor chain becomes insoluble. By using *b* = 0.5 nm and *s*_max_ = 0.25, we estimate *v*_d0_= −2 nm^3^ from eq 11.

Figure 2 shows the evolution of the size and conformation of a family of precursors with *b* = 0.5 nm, *N* = 500, and *v*_d0_= −2 nm^3^ as a function of the fraction of zwitterionic monomers in the precursor. Therefore, dipolar interactions allow tuning of the degree of compaction of the precursor chain containing *xN* reactive monomers for the subsequent formation of single-chain nanoparticles through the fraction of zwitterionic monomers incorporated into the precursor.

**Figure 2:**
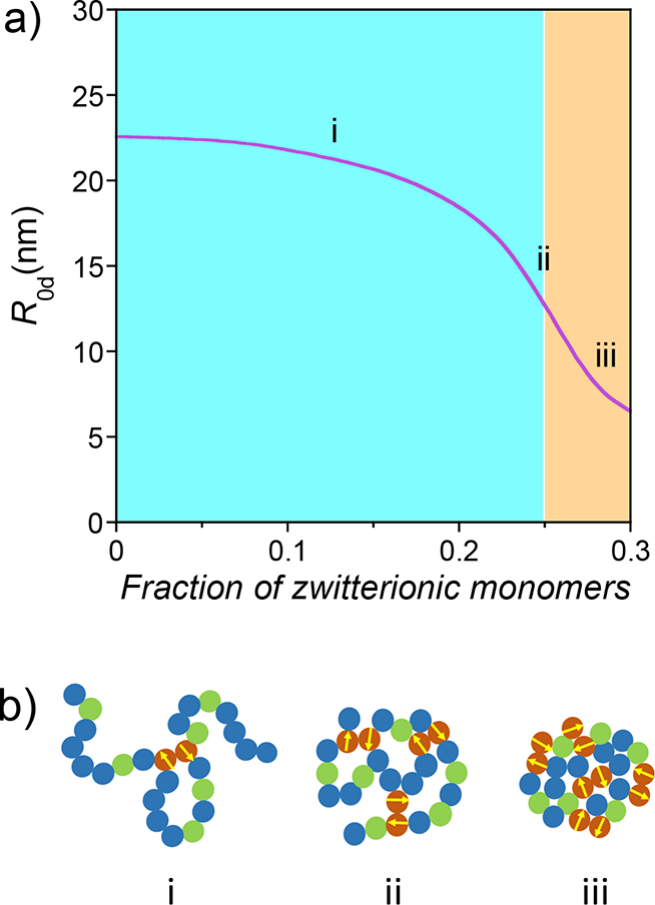
(a) Size (*R*_0d_) and conformation (i = self-avoiding, ii = Gaussian, iii = globular) of a family of precursors of single-chain nanoparticles (SCNPs) with *b* = 0.5 nm, *N* = 500, and *v*_d0_= −2 nm^3^ in a good solvent at high dilution without added salt as a function of the fraction of zwitterionic monomers in the precursor (*s*), as calculated from eq 7. (b) Cartoon of the evolution of the conformation of a precursor chain upon increasing *s* (see the text for details).

#### 2.1.2 Precursor in a Good Solvent at High Dilution with Added Salt

The dipole–dipole interaction weakens with an increase in the added salt concentration due to screening of the attractive dipolar forces. According to Muthukumar,^76^ in the case of quenched quadrupoles, *v*_d_ becomes

$$v_{d}=v_{d0}(1+\kappa r_{0})e^{-\kappa r_{0}}$$ 12

where κ^–1^ (nm) $≅0.3/\sqrt{c_{s}}$ for monovalent salts in water (*c*_s_ in units of moles per liter, M) and *r*_0_ is the average distance between adjacent dipoles.^80^ Eq 12 does not take into account collective charge density fluctuations that could play a dominant role at very high salt concentrations.^81^ Moreover, eq 12 is presumably valid only when the Debye screening length is higher than the distance between adjacent dipoles (i.e., κ^–1^ > *r*_0_) because otherwise the opposite charges of the dipole can interact as effectively disconnected charges.^81^ Accordingly, eq 7 becomes

$$\frac{2R_{0d}}{b^{2}N}-3(b^{3}+s^{2}v_{d})\frac{N^{2}}{R_{0d}^{4}}-6b^{6}\frac{N^{3}}{R_{0d}^{7}}=0$$ 13

The size of a family of precursors with *b* = 0.5 nm, *N* = 500, *v*_d0_ = −2 nm^3^, and *r*_0_ = 0.25 nm^76^ as a function of *c*_s_ is illustrated in Figure 3. The increase in size of polyzwitterion chains upon increasing the salt concentration is called the “anti-polyelectrolyte” behavior.^48^ Upon increasing *c*_s_, |*v*_d_| decreases and *s*_max_ shifts to higher values according to

$$s_{\max}={\left(-\frac{b^{3}}{v_{d}}\right)}^{1/2}$$ 14

**Figure 3:**
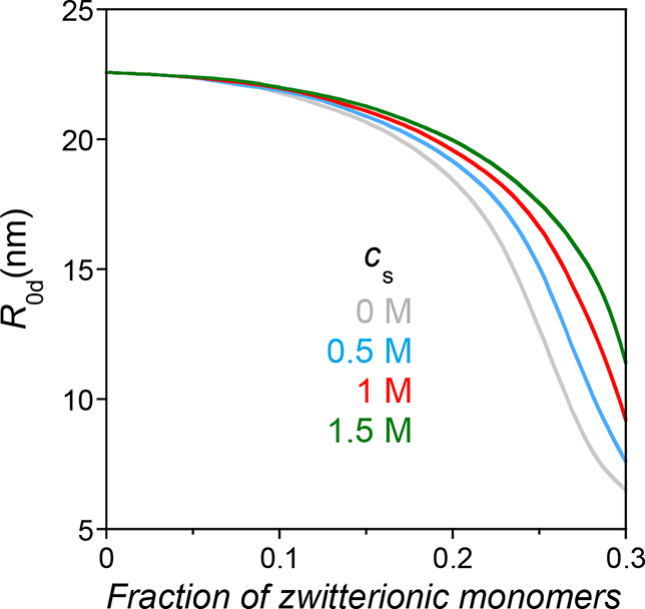
Illustration of the size (*R*_0d_) of a family of precursors with *b* = 0.5 nm, *N* = 500, *v*_d0_ = −2 nm^3^, and *r*_0_ = 0.25 nm as a function of the concentration of added salt, *c*_s_, and the fraction of zwitterionic monomers in the precursor, as calculated from eqs 12 and 13 (see the text for details).

For instance, we estimate the value of *s*_max_ as 0.27, 0.28, and 0.29 at *c*_s_ = 0.5, 1, and 1.5 M, respectively, from eqs 12 and 14 with *b* = 0.5 nm, *v*_d0_ = −2 nm^3^, and *r*_0_ = 0.25 nm, whereas *s*_max_ = 0.25 for *c*_s_ = 0 M.

#### 2.1.3 Summary of the Size and Conformation of a Linear Precursor of SCNPs with Intrachain Dipolar Interactions between Zwitterionic Monomers in a Diluted Solution

Table 1 provides a summary of the size and conformation of a linear precursor of SCNPs with dipolar interactions between *sN* zwitterionic monomers in a very good solvent at high dilution (*c* < c*) as a function of the balance between dipolar forces (*s* ^2^ *v*_d_) and excluded volume interactions (*v*_ev_ = *b* ^3^).

**Table 1:** Size and Conformation of a Linear Precursor of SCNPs with Intrachain Dipolar Interactions between Zwitterionic Monomers in a Very Good Solvent at High Dilution as a Function of the Balance between Dipolar Forces and Excluded Volume Interactions^a^

balance of interactions	size	conformation (scaling exponent, ν)
dipolar ≪ excluded volume |*s* ^2^ *v*_d_| ≪ *b* ^3^	$R_{0d}\approx b{\left(1+\frac{s^{2}v_{d}}{b^{3}}\right)}^{1/5}N^{3/5}$	self-avoiding walk (ν = 3/5)
dipolar = excluded volume |*s* ^2^ *v*_d_| = *b* ^3^	$R_{0d}\approx {bN}^{1/2}$	random walk (ν = 1/2)
dipolar ≫ excluded volume |*s* ^2^ *v*_d_| ≫ *b* ^3^	$R_{0d}\approx b{\left(\left|1+\frac{s^{2}v_{d}}{b^{3}}\right|\right)}^{-1/3}N^{1/3}$	globular (ν = 1/3)

a *R*_0d_, size of the precursor with intrachain dipolar interactions (*R*_0d_ ∝ *N* ^*v*^); *b,* monomer size; *s,* fraction of zwitterionic monomers; *v*_d_= *v*_d0_(1 + κ*r*_0_)­e^–κ*r*_0_^, where *v*_d0_ is the strength of the dipolar interactions in the absence of added salt, κ is the inverse Debye length, *r*_0_ is the separation distance between dipoles; *N,* total number of monomers in the precursor; and c*= *N*/*R*_0d_^3^ (see the text for details).

### 2.2 Size, Conformation, and Local Domain Size of SCNPs with Intrachain Covalent and Dipolar Interactions in a Diluted Solution

To investigate the size (*R*_1d_) and conformation of SCNPs with covalent and dipolar interactions at high dilution ( $c<c^{*}=\frac{N}{R_{1d}^{3}}$ ) in a good solvent (see Figure 4), we combine the standard ESN model of neutral (nonamphiphilic) SCNPs^40,42–44^ with the MFD theory of polyzwitterion solutions.^76^ Additionally, we estimate the local domain size and number of monomers per domain of SCNPs with intrachain covalent and dipolar interactions by means of the scaling approach described in ref 41. We consider the cases of an isolated SCNP in a good solvent at high dilution, both with and without added salt. For the case *c* > c*, see the SI.

**Figure 4:**
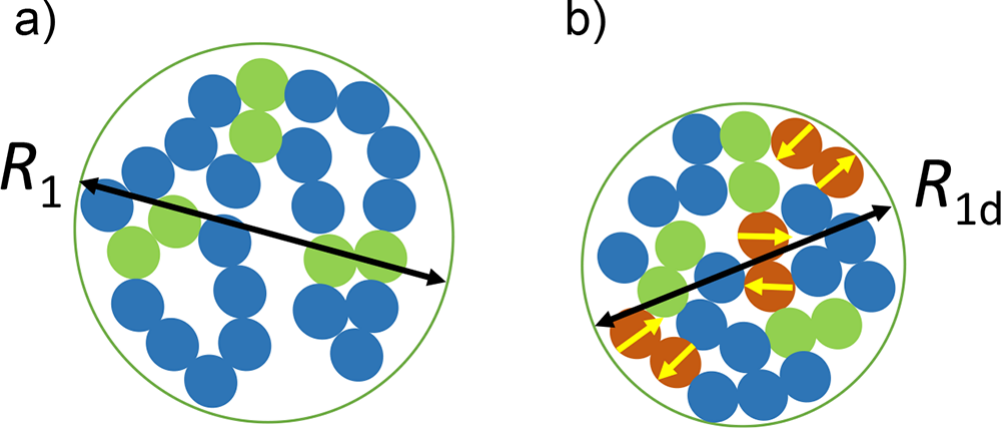
Cartoon to illustrate the effect of intrachain interactions on the size of an isolated SCNP in solution at high dilution (without added salt): (a) with only intrachain covalent bonds between reactive monomers (green color) showing size *R*_1_, and (b) with both intrachain covalent bonds and dipole–dipole interactions (yellow arrows) between zwitterionic monomers (brown color) showing size *R*_1d_.

#### 2.2.1 SCNPs with Intrachain Covalent and Dipolar Interactions in a Good Solvent at High Dilution (No Added Salt): Size and Conformation

The free energy of an SCNP prepared from a precursor with a fraction of reactive monomers *x* and a fraction of zwitterionic monomers *s* (see Section 2.1) contains the same contributions as shown in eq 1. However, according to the ESN model,^40,42–44^ the stretching contribution to the free energy (*F*_1_) is now given by

$${\beta F}_{1}\approx KR^{2}+\frac{R^{2}}{b^{2}N}$$ 15

where *K* = *Ax* and *A* is the SCNP elasticity constant. The second term on the r.h.s. of eq 15 is negligible inasmuch *K* = *Ax* ≫(*b* ^2^ *N*)^−1^, which is usually the case unless *x* is extremely low. The contributions from binary (*F*_2_) and ternary (*F*_3_) excluded volume interactions as well as dipole–dipole (*F*_4_) forces are still given by eqs 3–5, respectively. Accordingly, the free energy of an isolated SCNP with covalent and dipolar interactions between zwitterionic monomers in a good solvent at high dilution becomes

$$\beta F\approx KR^{2}+v_{\mathrm{eff}}\frac{N^{2}}{R^{3}}+b^{6}\frac{N^{3}}{R^{6}}$$ 16

with *v*_eff_ ≡ *b* ^3^ + *s* ^2^ *v*_d0_. In writing eq 16, we implicitly assume that all reactive monomers have reacted, forming intrachain cross-linking points, and also that a number of dipole–dipole interactions equivalent to that in the (unreacted) precursor chain has been established (see Figure 1B). Minimization of eq 16 with respect to *R* provides an expression to calculate the value of the equilibrium size of the SCNP with both intrachain covalent bonds and dipole–dipole interactions (*R*_1d_):

$$2KR_{1d}-3(b^{3}+s^{2}v_{d0})\frac{N^{2}}{R_{1d}^{4}}-6b^{6}\frac{N^{3}}{R_{1d}^{7}}=0$$ 17

As in the case of the precursor chain, three limiting scaling laws can be obtained from eq 17 depending on the balance between attractive dipolar forces (*s* ^2^ *v*_d0_ < 0) and repulsive excluded volume interactions (*v*_ev_ = *b* ^3^ > 0):

- If |*s* ^2^ *v*_d0_| ≪ *b* ^3^, we obtain

$$R_{1d}\approx b^{3/5}{\left(1+\frac{s^{2}v_{d0}}{b^{3}}\right)}^{1/5}K^{-1/5}N^{2/5}$$ 18

For *s* = 0 (i.e., no zwitterionic monomers in the precursor chain), eq 18 reduces to the expression of the size of SCNPs without dipolar interactions (*R*_1_ ≈ *b* ^3/5^ *K* ^–1/5^ *N* ^2/5^).^40,42–44^ Experimental values of canonical SCNPs investigated through SAXS measurements and MD simulations^82^ and SCNPs from the partially denatured BSA protein^83^ provided values of the size scaling exponent in good agreement with the theoretical value of 2/5 and within the combined uncertainties of the experimental techniques and the theoretical approach (i.e., ν = 2/5 for *N* → ∞). Also, the scaling exponent for *K* (−1/5) is supported by both experimental data and MD simulation data of SCNPs from common precursor polymers within acceptable error limits (−0.20 and −0.22, respectively).^41,44^ The relative size reduction in an isolated SCNP due to the presence of dipolar interactions (i.e., *R*_1d_/*R*_1_) is

$$\frac{R_{1d}}{R_{1}}\approx {\left(1+\frac{s^{2}v_{d0}}{b^{3}}\right)}^{1/5}$$ 19

which is identical to eq 9 corresponding to the relative size reduction in a precursor chain due to the presence of dipolar interactions (i.e., *R*_0d_/*R*_0_).

- If |*s* ^2^ *v*_d0_| = *b* ^3^, the SCNP adopts the conformation of an isolated SCNP in a theta solvent^40,42–44^ (*R*_1d_ ≈ *b* ^3/4^ *K* ^–1/8^ *N* ^3/8^). The “ideal” (theta solvent, melt) size scaling exponent (ν = 3/8) of SCNPs is also in good agreement with the experimental values of canonical SCNPs as determined by SAXS measurements and MD simulations (0.37).^82^ Collectively, the good agreement between theoretical and experimental scaling exponents for SCNPs under ideal and good solvent conditions supports the validity of the ESN model.
- If |*s* ^2^ *v*_d0_| ≫ *b* ^3^, the conformation of the SCNP becomes globular (*R*_1d_ ∝ *N* ^1/3^) with a size given by eq 10. Now, *R*_1d_ is controlled exclusively by the balance of the effective second virial and third virial coefficients, which are identical for the precursor and SCNPs. This means that no size change can be expected when intrachain cross-linking of the precursor is performed under these conditions.

Figure 5 illustrates the change in size and conformation of a family of SCNPs with *b* = 0.5 nm, *N* = 500, *A* = 15 nm^–2^, and *v*_d0_= −2 nm^3^ as a function of the fraction of zwitterionic monomers (*s*) and the fraction of intrachain cross-linked monomers (*x*). In Figure 5, the most notorious reduction in size upon increasing the fraction of zwitterionic monomers in the SCNP is observed for the SCNPs with a lower fraction of intrachain cross-linked monomers (i.e., *x* = 0.05).

**Figure 5:**
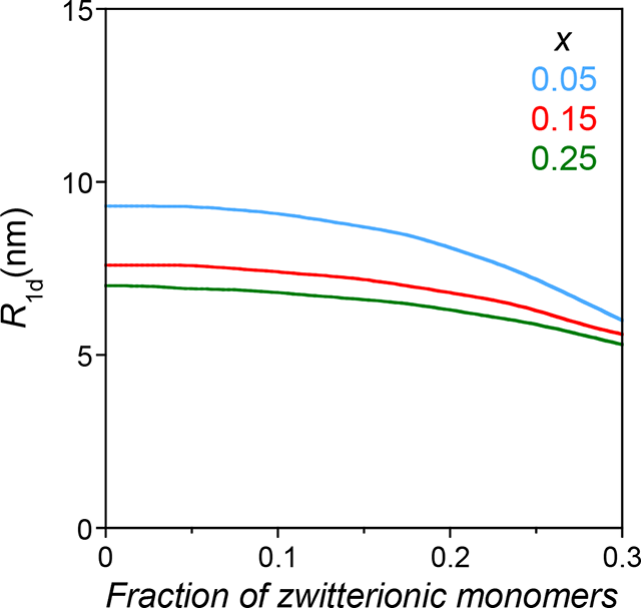
Illustration of the size (*R*_1d_) of a family of SCNPs with *b* = 0.5 nm, *N* = 500, *A* = 15 nm^–2^, and *v*_d0_ = −2 nm^3^ as a function of the fraction of zwitterionic monomers for different values of the fraction of intrachain cross-linked monomers (*x*), as calculated from eq 17 (see the text for details).

#### 2.2.2 SCNPs with Intrachain Covalent and Dipolar Interactions in a Good Solvent at High Dilution with Added Salt

In this case, eq 17 becomes

$$2KR_{1d}-3(b^{3}+s^{2}v_{d})\frac{N^{2}}{R_{1d}^{4}}-6b^{6}\frac{N^{3}}{R_{1d}^{7}}=0$$ 20

with *v*_*d*_ given by eq 12 in the case of quenched quadrupoles,^76^ and κ^–1^ (nm) $≅0.3/\sqrt{c_{s}}$ for monovalent salts in water.^80^

The size (*R*_1d_) of a family of SCNPs with *b* = 0.5 nm, *N* = 500, *A* = 15 mn^–2^, *x* = 0.05, *v*_d0_ = −2 nm^3^, and *r*_0_ = 0.25 nm^76^ as a function of *c*_s_ is illustrated in Figure 6. The “anti-polyelectrolyte” behavior is also observed for the case of SCNPs with dipolar interactions in a diluted solution with added salt. Upon increasing the *c*_s_ from 0 to 0.75 and 1.5 M, the value of *s*_max_ grows from 0.25 to 0.27 and 0.29, respectively, as estimated from eq 14.

**Figure 6:**
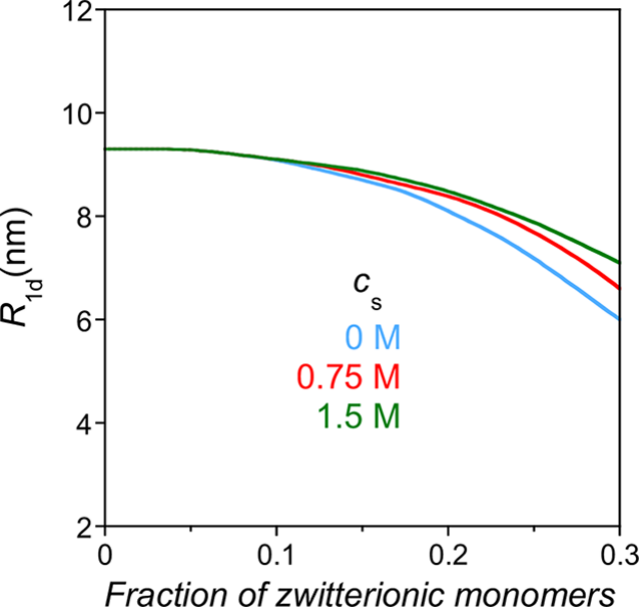
Illustration of the size (*R*_1d_) of a family of SCNPs with *b* = 0.5 nm, *N* = 500, *A* = 15 mn^–2^, *x* = 0.05, *v*_d0_ = −2 nm^3^, and *r*_0_ = 0.25 nm as a function of the concentration of added salt, *c*_s_, and the fraction of zwitterionic monomers in the precursor as calculated from eqs 12 and 20 (see the text for details).

#### 2.2.3 Local Domain Size and Number of Monomers per Domain

The local domain size (ξ_d_) and the number of monomers per domain (*g*_d_) in SCNPs with intrachain and dipolar interactions can be obtained by following the scaling approach described in ref 41. Essentially, the free energy of folding and collapse is first estimated by considering the confinement of a chain of size *R*_0d_ in a cavity of size *R*_1d_. Next, this free energy is assumed to be proportional to the ratio of the volume of the SCNP ( $\propto {R_{1d}}^{3}$ ) to the volume of each local domain ( $\propto {\xi_{d}}^{3}$ ). This procedure gives the number of local domains with an energy around *k*_B_ *T* each (as usual in scaling analysis) and, hence, allows one to estimate ξ_d_ and *g*_d_, such as

$$\xi_{d}\approx R_{1d}{\left(\frac{R_{1d}}{R_{0d}}\right)}^{-1/3\delta }$$ 21

$$g_{d}\approx N{\left(\frac{\xi_{d}}{R_{1d}}\right)}^{3}$$ 22

where δ is the scaling exponent of *R*_1d_ on *x* (i.e., *R*_1d_ ∝ *K* ^δ^ ∝ *x* ^δ^). Consequently,

- For SCNPs with attractive dipolar interactions much weaker than repulsive excluded volume interactions (i.e., |*s* ^2^ *v*_d0_| ≪ *b* ^3^), *R*_1d_ and *R*_0d_ are given by eqs 18 and 8, respectively, so $\delta =-\frac{1}{5}$ . Hence, we obtain

$$\xi_{d}\approx R_{1d}{\left(\frac{R_{1d}}{R_{0d}}\right)}^{5/3}$$ 23

$$g_{d}\approx N{\left(\frac{R_{1d}}{R_{0d}}\right)}^{5}$$ 24

eq 23 provides the size of the local compact domains of SCNPs in solution, which can differ slightly from the domain size observed by high-resolution transmission electron microscopy in the dry state.^84^

- For SCNPs in which the attractive dipolar forces balance with the repulsive excluded volume ones (i.e., |*s* ^2^ *v*_d0_| = *b* ^3^), $R_{1d}\approx b^{3/4}K^{-1/8}N^{3/8}$ so $\delta =-\frac{1}{8}$ and we have

$$\xi_{d}\approx R_{1d}{\left(\frac{R_{1d}}{R_{0d}}\right)}^{8/3}$$ 25

$$g_{d}\approx N{\left(\frac{R_{1d}}{R_{0d}}\right)}^{8}$$ 26

- However, for SCNPs with |*s* ^2^ *v*_d0_| ≫ *b* ^3^, we have δ = 0, so eqs 20 and 21 cannot be used to estimate the size and number of local compact domains, since these expressions are only valid for δ ≠ 0.

### 2.3 Size and Conformation of a Linear Precursor of SCNPs with Intrachain Dipolar and Electrostatic Interactions in Solution

We consider now a precursor composed of *N* monomers per chain, where *x* is the fraction of reactive monomers, *f* is the fraction of monomers carrying the elemental electrical charge, *s* is the fraction of zwitterionic monomers, and, consequently, 1–*x*–*f*–*s* is the fraction of inert monomers. We assume that the reactive, charged, and zwitterionic monomers are distributed in a random fashion along the chain. Furthermore, we expect *f* to be above a threshold value, *f*_min_, that is determined below.

#### 2.3.1 Precursor with Intrachain Dipolar and Electrostatic Interactions in a Good Solvent at High Dilution (No Added Salt)

In a highly diluted salt-free solution, according to the classical scaling theory of weak PEs (without logarithmic corrections) developed by Dobrynin et al.,^45^ the presence of long-range (repulsive) electrostatic interactions between charged monomers along the precursor induces significant stretching of the chain. We assume all counterions have been released to the solution, so counterion (i.e., Manning) condensation effects are absent.^46^ This is a very good approximation for a highly dilute PE solution with *fu* ^2^ < 1 (i.e., weak-PE condition),^46^ where $u≡\frac{l_{B}}{b}$ (*l*_*B*_ is the Bjerrum length). As depicted in Figure 7, the precursor adopts an extended conformation of electrostatic blobs of size *D*_e_ and number of monomers per blob *g*_e_ with the precursor size (*R*_0de_) given by^45^

$$R_{0\mathrm{de}}\approx D_{e}\left(\frac{N}{g_{e}}\right)$$ 27

**Figure 7:**
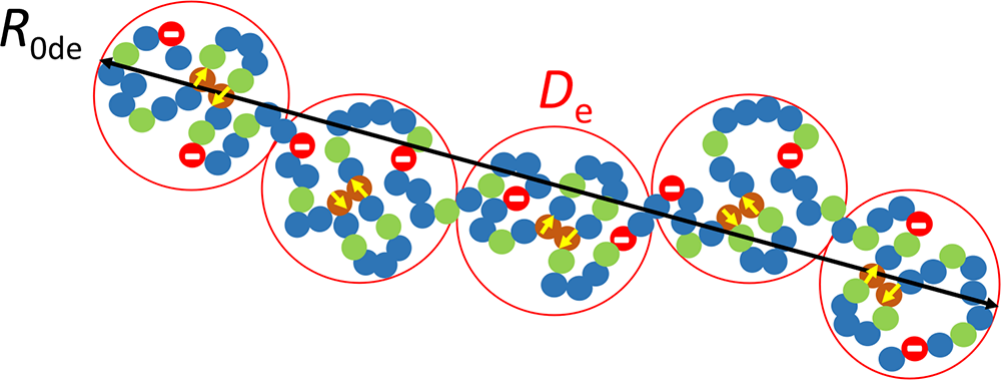
Cartoon to illustrate the extended conformation of a precursor chain of total number of monomers *N* in a salt-free diluted solution containing *xN* reactive monomers (green color), *fN* charged monomers involved in long-range electrostatic interactions between them (red color), *sN* zwitterionic monomers (brown color) with pairwise dipole–dipole interactions to give quadrupoles (yellow arrows), and (1–*x*–*f*–*s*)*N* inert monomers (blue color). *R*_0de_ is the precursor size, and *D*_e_ is the size of the electrostatic blobs.

The chain stretching is a consequence of the strong repulsion between the electrostatic blobs. *D*_e_ can be estimated by equating the electrostatic energy of each blob with the thermal energy:^45^

$$k_{B}T\left(\frac{e^{2}(fg_{e}{)}^{2}}{\varepsilon D_{e}}\right)=k_{B}T\left(\frac{bu(fg_{e}{)}^{2}}{D_{e}}\right)\approx k_{B}T$$ 28

where *e* and ε are the elemental charge and the dielectric constant of the medium, respectively. Inside the blob (*r* < *D*_e_), the conformation of the *g*_e_ monomers within the blob will be that of a precursor with dipolar interactions free from electrostatic interactions (see Section 2.2). For a very good solvent (*v*_ev_ = *b* ^3^ ≠ 0), we can consider three cases depending on the balance between attractive–dipolar–interactions (*s* ^2^ *v*_d0_) and repulsive–excluded volume–effects (*v*_ev_ = *b* ^3^):

- If |*s* ^2^ *v*_d0_| ≪ *b* ^3^, we have (see Table 1)

$$D_{e}\approx b{\left(1+\frac{s^{2}v_{d0}}{b^{3}}\right)}^{1/5}g_{e}^{3/5}$$ 29

By solving eqs 28 and 29, we obtain

$$g_{e}\approx {\left(1+\frac{s^{2}v_{d0}}{b^{3}}\right)}^{1/7}u^{-5/7}f^{-10/7}$$ 30

$$D_{e}\approx b{\left(1+\frac{s^{2}v_{d0}}{b^{3}}\right)}^{2/7}u^{-3/7}f^{-6/7}$$ 31

and by substituting eqs 30 and 31 in eq 27, we have

$$R_{0\mathrm{de}}\approx b{\left(1+\frac{s^{2}v_{d0}}{b^{3}}\right)}^{1/7}Nu^{2/7}f^{4/7}$$ 32

Importantly, eqs 30–32 reduce to eqs 4, 6, and 7 of ref 45 (case: *T* ≫ θ) when *s* or *v*_d0_ → 0, as expected. Eq 30 can be used to establish the range of *f* in which eqs 30–32 are valid. Hence, the minimum value of *f* would be that in which *g*_e_ is equal to *N* (i.e., all of the monomers are contained within a single electrostatic blob):

$$f_{\min}\approx {\left(1+\frac{s^{2}v_{d0}}{b^{3}}\right)}^{1/10}N^{-7/10}u^{-1/2}$$ 33

whereas the maximum value of *f* can be estimated from the weak-PE condition (*fu* ^2^ < 1):^46^

$$f_{\max}\approx u^{-2}$$ 34

For instance, for a precursor with *b* = 0.5 nm, *s* = 0.1, *v*_d0_= −2 nm^3^, *u* = 1.4, and *N* = 500, the model is presumably valid only in the range 0.01 < *f* < 0.51, as estimated from eqs 33 and 34. Importantly, for *f* ≤ *f*_min_, the size and conformation of the precursor are given by eq 8 (SAW conformation) instead of eq 32 (extended conformation). That means that a small amount of electrostatic charges is tolerated in a self-avoiding precursor chain before significant electrostatic stretching effects become operative (e.g., a precursor chain of *N* = 500 and *f*_min_ = 0.01 contains 5 charged monomers). As usual, we can obtain an indication of how far the precursor size is from that of a fully rigid rod of size *bN* through the stretching parameter Ψ defined as Ψ ≡ *bN/R*_0de_.^45^ From eq 32, we obtain

$$\Psi \approx {\left(1+\frac{s^{2}v_{d0}}{b^{3}}\right)}^{-1/7}u^{-2/7}f^{-4/7}$$ 35

- If |*s* ^2^ *v*_d0_| = *b* ^3^, it can be shown that *g*_e_, *D*_e_, and *R*_0de_ are given by eqs[Disp-formula eq4], 6, and 7 of ref 45 (case: *T* = θ):

$$g_{e}\approx u^{-2/3}f^{-4/3}$$ 36

$$D_{e}\approx bu^{-1/3}f^{-2/3}$$ 37

$$R_{0\mathrm{de}}\approx bNu^{1/3}f^{2/3}$$ 38

In this case, the model would be valid in the range $f_{\min}=u^{-1/2}N^{-3/4}$ < *f* < *f*_max_ = *u* ^–2^. For *f* < *f*_min_, the size is given by $R_{0\mathrm{de}}\approx {bN}^{1/2}$ (see Table 1) instead of eq 38. Now, the parameter Ψ is given by

$$\Psi \approx u^{-1/3}f^{-2/3}$$ 39

- If |*s* ^2^ *v*_d0_| ≫ *b* ^3^, according to Dobrynin, Rubinstein, and Obukhov,^85^ we can expect the formation of a necklace globule with compact beads joined by narrow strings since this extended conformation has lower free energy than that of a cylindrical globule.^86–90^ The number of monomers in a bead can be estimated from^85^

$$g_{e}\approx \tau u^{-1}f^{-2}=|1+\frac{s^{2}v_{d0}}{b^{3}}|u^{-1}f^{-2}$$ 40

where in our case $\tau \approx \frac{\left|v_{\mathrm{eff}}\right|}{b^{3}}=|1+\frac{s^{2}v_{d0}}{b^{3}}|$ , and the diameter of the beads (size of the electrostatic blob) is given by eq 37. The total length of the necklace is^85^

$$R_{0\mathrm{de}}\approx b{\left(\frac{u}{\tau }\right)}^{1/2}fN=b{\left(\left|1+\frac{s^{2}v_{d0}}{b^{3}}\right|\right)}^{-1/2}Nu^{1/2}f$$ 41

In this case, the model will presumably be valid in the range $f_{\min}={\left(\left|1+\frac{s^{2}v_{d0}}{b^{3}}\right|\right)}^{1/2}N^{-1/2}u^{-1/2}$ , corresponding to *g*_e_ = *N* (i.e., a single bead), < *f* < *f*_max_ = *u* ^–2^. For *f* < *f*_min_, the size is given by eq 10 instead of eq 41. The parameter Ψ is given by

$$\Psi \approx {\left(\left|1+\frac{s^{2}v_{d0}}{b^{3}}\right|\right)}^{1/2}u^{-1/2}f^{-1}$$ 42

Since the number of beads, *N*_bead_, of the necklace globule can only be an integer, there are a set of boundaries between states of the necklace globule with different numbers of beads, each one taking place at a critical charge fraction, *f*_c_, according to^85^

$$f_{c}\approx {\left(|1+\frac{s^{2}v_{d0}}{b^{3}}|N_{\mathrm{bead}}\right)}^{1/2}N^{-1/2}u^{-1/2}\;\mathrm{where}\;N_{\mathrm{bead}}=1,2,3,\cdot \cdot \cdot$$ 43

Remarkably, *f*_c_ from eq 43 for *N*_bead_ = 1 gives ${\left(\left|1+\frac{s^{2}v_{d0}}{b^{3}}\right|\right)}^{1/2}N^{-1/2}u^{-1/2}=f_{\min}$ , as obtained above from eq 40 for *g*_e_ = *N* (i.e., a single bead).

Figure 8 shows a plot of *R*_0de_ vs *f* for a linear precursor of SCNPs with dipolar and electrostatic interactions in a good solvent at high dilution without added salt as a function of the balance between dipolar interactions and excluded volume effects.

**Figure 8:**
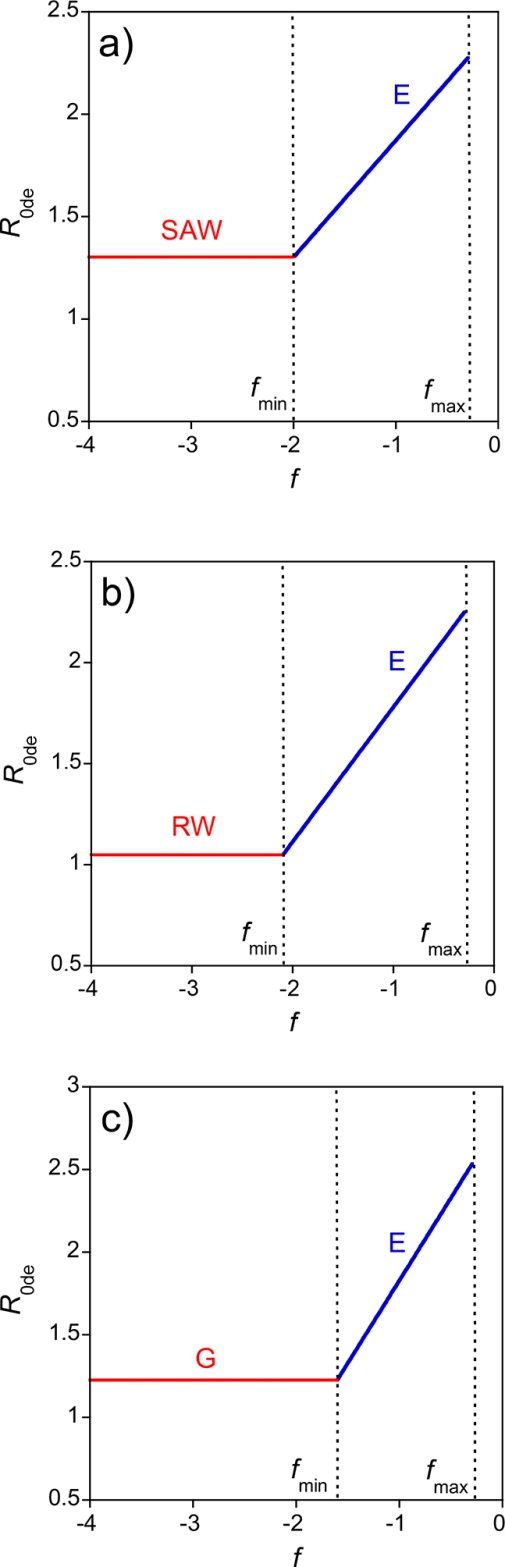
*R*_0de_ vs *f* (Log–Log plot) for a linear precursor of SCNPs with dipolar and electrostatic interactions in a good solvent at high dilution (*b* = 0.5 nm, *v*_d0_= −2 nm^3^, *u* = 1.4, and *N* = 500) depending on the balance between attractive–dipolar–interactions and repulsive–excluded volume–effects: (a) *s* = 0.1, |*s* ^2^ *v*_d0_| ≪ *b* ^3^; (b) *s* = 0.25, |*s* ^2^ *v*_d0_| = *b* ^3^; and (c) *s* = 0.3, |*s* ^2^ *v*_d0_| ≫ *b* ^3^. Conformation denoted as SAW, self-avoiding walk; RW, random walk; G, globular; and E, extended. Weak-PE regime: *f*_min_ < *f* < *f*_max_ (see the text for details).

#### 2.3.2 Summary of the Size and Conformation of a Linear Precursor of SCNPs with Intrachain Dipolar and Electrostatic Interactions in a Good Solvent (No Added Salt)

A summary of the size and conformation of a linear precursor of SCNPs with dipolar and electrostatic interactions in a very good solvent at high dilution (without added salt) as a function of the balance between dipolar forces and excluded volume interactions is given in Table 2. For completeness, we additionally include the results corresponding to semidiluted conditions, which are more relevant for comparison with experimental data (see the Supporting Information, SI).

**Table 2:** Size and Conformation of a Linear Precursor of SCNPs with Dipolar and Electrostatic Interactions in a Very Good Solvent (No Added Salt) as a Function of the Balance between Dipolar Forces and Excluded Volume Interactions^a^

balance of interactions	size^b^	conformation (scaling exponent, ν)
highly diluted conditions (c < c*)		
dipolar ≪ excluded volume |*s* ^2^ *v*_d0_| ≪ *b* ^3^	$R_{0\mathrm{de}}\approx b{\left(1+\frac{s^{2}v_{d0}}{b^{3}}\right)}^{1/7}Nu^{2/7}f^{4/7}$	extended (ν = 1)
dipolar = excluded volume |*s* ^2^ *v*_d0_| = *b* ^3^	$R_{0\mathrm{de}}\approx bNu^{1/3}f^{2/3}$	extended (ν = 1)
dipolar ≫ excluded volume |*s* ^2^ *v*_d0_| ≫ *b* ^3^	$R_{0\mathrm{de}}\approx b{\left(\left|1+\frac{s^{2}v_{d0}}{b^{3}}\right|\right)}^{-1/2}Nu^{1/2}f$	extended^d^ (ν = 1)
semidiluted conditions (c* < c < c**)^e^		
dipolar ≪ excluded volume |*s* ^2^ *v*_d0_| ≪ *b* ^3^	$R_{c0\mathrm{de}}\approx b^{1/4}{\left(1+\frac{s^{2}v_{d0}}{b^{3}}\right)}^{1/28}N^{1/2}u^{1/14}f^{1/7}c^{-1/4}$	random walk (ν = 1/2)
dipolar = excluded volume |*s* ^2^ *v*_d0_| = *b* ^3^	$R_{c0\mathrm{de}}\approx b^{1/4}N^{1/2}u^{1/12}f^{1/6}c^{-1/4}$	random walk (ν = 1/2)
dipolar ≫ excluded volume |*s* ^2^ *v*_d0_| ≫ *b* ^3^	$R_{c0\mathrm{de}}\approx b^{1/4}{\left(\left|1+\frac{s^{2}v_{d0}}{b^{3}}\right|\right)}^{-1/8}N^{1/2}u^{1/8}f^{1/4}c^{-1/4}$	random walk^d^ (ν = 1/2)

a *R*_0de_, size of the precursor with intrachain dipolar and electrostatic interactions (*R*_0de_ ∝ *N* ^*v*^) at *c* < c*; *b,* monomer size; *s,* fraction of zwitterionic monomers; *v*_d0_, strength of the dipolar interactions in the absence of added salt; *u,* *l*_B_/*b* (*l*_B_ is the Bjerrum length); *f*, fraction of charged monomers; *N,* total number of monomers; and *R*_c0de_, size of the precursor with intrachain dipolar and electrostatic interactions at c*< *c* < c**.

b *f*_min_ < *f* < *f*_max_ (see the text for details).

^*c*^Here, $c^{*}=\frac{N}{R_{0\mathrm{de}}^{3}}$

d Necklace globule conformation.

e See the SI.

#### 2.3.3 Precursor with Intrachain Dipolar and Electrostatic Interactions in a Good Solvent at High Dilution with Added Salt

Let us assume that the concentration of the precursor in solution, *c*, is substantially below the overlap concentration, c*, given by

$$c^{*}\approx \frac{N}{(R_{0\mathrm{de}}{)}^{3}}$$ 44

According to the scaling theory of PE solutions,^45^ if the concentration of the (monovalent) salt in solution, *c*_s_, is higher than a critical value, *c*_s_^*^, given by

$$c_{s}^{*}\approx \frac{1}{N^{2}}{\left(\frac{\Psi }{b}\right)}^{3}\left(\frac{f}{2}\right)$$ 45

then the precursor will become an SAW of electrostatic screening blobs with a size given by^45^

$$R_{0\mathrm{de}}\approx bN^{3/5}(cb^{3}{)}^{-1/5}\Psi^{-2/5}{\left(1+\frac{2c_{s}}{fc}\right)}^{-1/5}$$ 46

Implicit in eq 46 is a linear dependence of the electrostatic persistence length *l*_p_ on the Debye length κ^–1^. The dependence of *l*_p_ on κ^–1^ has been a controversial topic in the literature.^91,92^ The *l*_p_ α κ^–1^ relationship holds specially well at high salt concentrations and for flexible PEs^93^ as supported by several experimental^94,95^ and theoretical studies.^96,97^ Conversely, for locally stiff PEs^98^ (not considered here), eq 46 should be modified by taking into account a quadratic dependence of *l*_p_ on κ^–1^, according to the works of Odijk^99,100^ and Skolnick and Fixman.^101^

In the case of a linear precursor with dipolar and electrostatic interactions, eq 46 takes different forms depending on the balance between attractive–dipolar–interactions in the presence of a salt (*s* ^2^ *v*_d_) and repulsive–excluded volume–effects (*v*_ev_ = *b* ^3^), where *v*_d_ is now given by eq 12, and Ψ by eqs 35, 39, and 42 (with *v*_d0_ replaced by *v*_d_) for |*s* ^2^ *v*_d_| ≪ *b* ^3^, |*s* ^2^ *v*_d_| = *b* ^3^, and |*s* ^2^ *v*_d_| ≫ *b* ^3^, respectively.

Figure 9 shows a plot of *R*_0de_ vs *c*_s_ according to eq 46 for a linear precursor of SCNPs with dipolar and electrostatic interactions in a good solvent at high dilution as a function of the balance between dipolar interactions and excluded volume effects. It can be shown that in the range 10^–7^ < *c*_s_ < 10^–2^, *v*_d_ ∼ *v*_d0_ can be considered a very good approximation since in this range *v*_d_/*v*_d0_ = 0.999–0.997. As illustrated in Figure 9A for the case |*s* ^2^ *v*_d_| ≪ *b* ^3^, below *c*_s_^*^, the precursor maintains an extended PE conformation with size given by eq 32. For *c*_s_ > *c*_s_^*^, the conformation changes to an SAW and progressively reduces according to eq 46 toward the limiting size of a SAW precursor with dipolar interactions, as given by eq 8. A subsequent increase in *c*_*s*_ increases the size of the SAW precursor, although very slowly, due to the weakening of dipole–dipole interactions according to eq 13. A similar behavior is observed when |*s* ^2^ *v*_d_| = *b* ^3^, but in this case, at a high salt concentration, the precursor adopts a limiting RW conformation and its size no longer changes by increasing the salt concentration (see Figure 9B). For the case |*s* ^2^ *v*_d_| ≫ *b* ^3^, upon increasing *c*_s_ above *c*_s_^*^, the precursor in an extended conformation progressively reduces its size through an SAW conformation and goes to a globular conformation at high salt concentrations.

**Figure 9:**
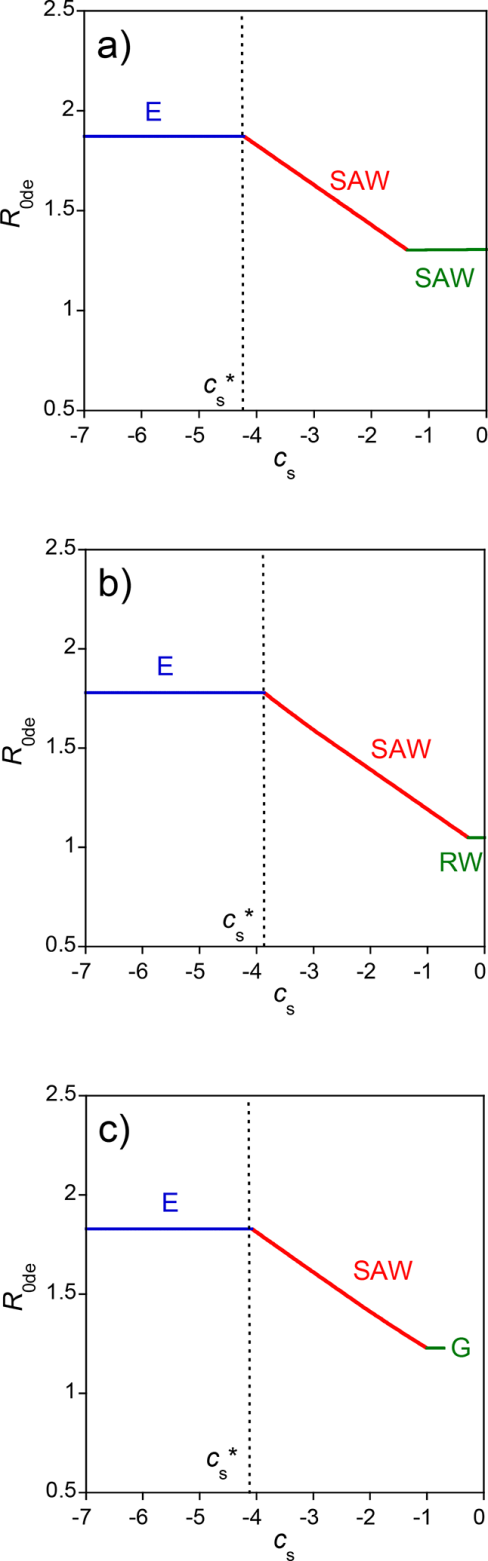
*R*_0de_ vs *c*_s_ (Log–Log plot) for a linear precursor of SCNPs with dipolar and electrostatic interactions in a good solvent at high dilution (*b* = 0.5 nm, *v*_d0_ = −2 nm^3^, κ^–1^ (nm) $≅\frac{0.3}{\sqrt{c_{s}}},\;r_{0}$ = 0.25 nm, *u* = 1.4, *f* = 0.1, *N* = 500, and *c* = 10^–5^ M) as a function of the balance between attractive–dipolar–interactions and repulsive–excluded volume–effects: (a) *s* = 0.1, |*s* ^2^ *v*_d0_| ≪ *b* ^3^; (b) *s* = 0.25, |*s* ^2^ *v*_d0_| = *b* ^3^; and (c) *s* = 0.3, |*s* ^2^ *v*_d0_| ≫ *b* ^3^. Conformation denoted as E, extended; SAW, self-avoiding walk; RW, random walk; and G, globular. Electrostatic interactions are only relevant for *c*_s_ < *c*_s_^*^ (see the text for details).

### 2.4 Size, Conformation, and Local Domain Size of SCNPs with Intrachain Covalent, Dipolar, and Electrostatic Interactions in a Diluted Solution

#### 2.4.1 SCNPs with Intrachain Covalent, Dipolar, and Electrostatic Interactions in a Good Solvent at High Dilution (No Added Salt)

We consider the formation of an isolated SCNP in a (salt-free) good solvent from a precursor chain with *xN* reactive, *sN* zwitterionic, *fN* (*f* > *f*_min_) charged, and (1–*x*–*f*–*s*)*N* inert monomers (Section 2.3, Figure 7). Due to the presence of electrostatic blobs, which repel each other, along the isolated precursor chain,^45^ intrachain cross-linking will only be possible inside each blob via pairwise coupling of reactive monomers to give SCNPs (see Figure 10). Consequently, the blob size will decrease from *D*_e_ to *D*_c_ (i.e., *D*_c_ < *D*_e_) for all of the electrostatic blobs along the precursor chain, so the SCNP size (*R*_1de_) will now be given by

$$R_{1\mathrm{de}}\approx D_{c}\left(\frac{N}{g_{c}}\right)$$ 48

where *D*_c_ and *g*_c_ are the size (i.e., local domain size) and number of monomers in a cross-linked electrostatic blob, respectively. On the one hand, *D*_c_ is determined by the requirement of electrostatic energy at *r* = *D*_c_ to be on the order of thermal energy:

$$k_{B}T\left(\frac{bu(fg_{c}{)}^{2}}{D_{c}}\right)\approx k_{B}T$$ 49

**Figure 10:**
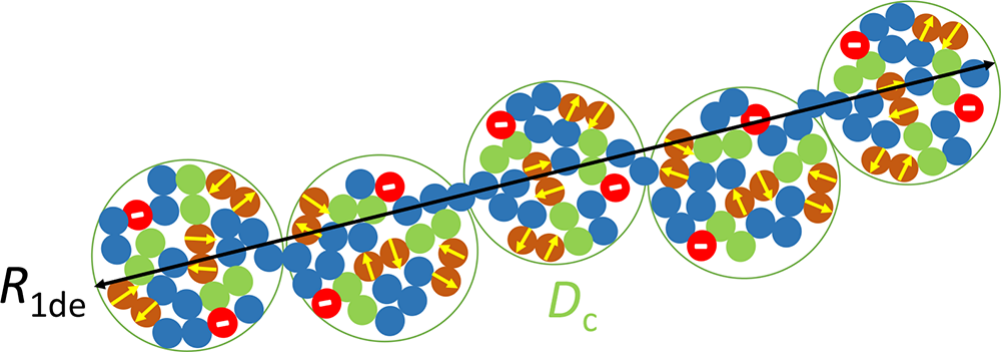
Cartoon to illustrate the conformation in solution at high dilution (without added salt) of an isolated SCNP with intrachain covalent bonds (green color), dipole–dipole interactions (yellow arrows) between zwitterionic monomers (brown color), and long-range electrostatic interactions (red color). *R*_1de_ is the SCNP size, and *D*_c_ is the cross-linked electrostatic blob size.

On the other hand, inside each cross-linked blob (*r* < *D*_c_), the conformation of the *g*_c_ monomers within each blob will be that of an SCNP with dipolar interactions free from electrostatic interactions (see Section 2.2). For a very good solvent (*v*_ev_ = *b* ^3^ ≠ 0), we can consider three cases depending on the balance between dipolar interactions (*s* ^2^ *v*_d0_) and excluded volume effects (*v*_ev_ = *b* ^3^).

- If |*s* ^2^ *v*_d0_| ≪ *b* ^3^, we have (see eq 18)

$$D_{c}\approx b^{3/5}{\left(1+\frac{s^{2}v_{d0}}{b^{3}}\right)}^{1/5}K^{-1/5}g_{c}^{2/5}$$ 50

By solving eqs 49 and 50, we obtain

$$g_{c}\approx b^{-1/4}{\left(1+\frac{s^{2}v_{d0}}{b^{3}}\right)}^{1/8}K^{-1/8}u^{-5/8}f^{-5/4}$$ 51

$$D_{c}\approx b^{1/2}{\left(1+\frac{s^{2}v_{d0}}{b^{3}}\right)}^{1/4}K^{-1/4}u^{-1/4}f^{-1/2}$$ 52

and by substituting eqs 51 and 52 in eq 48, we obtain

$$R_{1\mathrm{de}}\approx b^{3/4}{\left(1+\frac{s^{2}v_{d0}}{b^{3}}\right)}^{1/8}K^{-1/8}Nu^{3/8}f^{3/4}$$ 53

Importantly, eq 53 reduces to eq 32 of ref 47 when *s* or *v*_d0_ → 0, as expected. The expression for the parameter Ψ defined as Ψ ≡ *bN/R*_1de_ is

$$\Psi \approx b^{1/4}{\left(1+\frac{s^{2}v_{d0}}{b^{3}}\right)}^{-1/8}K^{1/8}u^{-3/8}f^{-3/4}$$ 54

- If |*s* ^2^ *v*_d0_| = *b* ^3^, *D*_c_ is given by (see Table 2)

$$D_{c}\approx b^{3/4}K^{-1/8}g_{c}^{3/8}$$ 55

By solving eqs 49 and 55, we obtain

$$g_{c}\approx b^{-2/13}K^{-1/13}u^{-8/13}f^{-16/13}$$ 56

$$D_{c}\approx b^{9/13}K^{-2/13}u^{-3/13}f^{-6/13}$$ 57

and by substituting eqs 56 and 57 in eq 48, we have

$$R_{1\mathrm{de}}\approx b^{11/13}K^{-1/13}Nu^{5/13}f^{10/13}$$ 58

Now, the parameter Ψ is given by

$$\Psi \approx b^{2/13}K^{1/13}u^{-5/13}f^{-10/13}$$ 59

- If |*s* ^2^ *v*_d0_| ≫ *b* ^3^, it can be shown that the expressions for *g*_c_, *D*_c_, *R*_1de_, and Ψ are given by eqs 37, 40–42, respectively.

Essentially, the characteristics of the necklace globule are controlled by the balance of Coulomb interactions and surface tension, which is identical for the precursor and SCNPs. Hence, no change in size is expected through intrachain cross-linking of the precursor to the SCNPs.

Figure 11 shows the size of SCNPs with intrachain covalent, dipolar, and electrostatic interactions in a very good solvent at high dilution (no added salt) as a function of the fraction of charged monomers (*f*), cross-linking degree (*x*) and balance between attractive dipolar interactions (*s* ^2^ *v*_d0_) and repulsive excluded volume effects (*v*_ev_ = *b* ^3^). Above *f*_min_, the conformation of the SCNPs is extended (*R*_1de_ ∝ *N*) in all of the cases. As explained before, when the attractive dipole–dipole forces become much stronger than the repulsive excluded volume effects, then *R*_1de_ becomes independent of the cross-linking degree.

**Figure 11:**
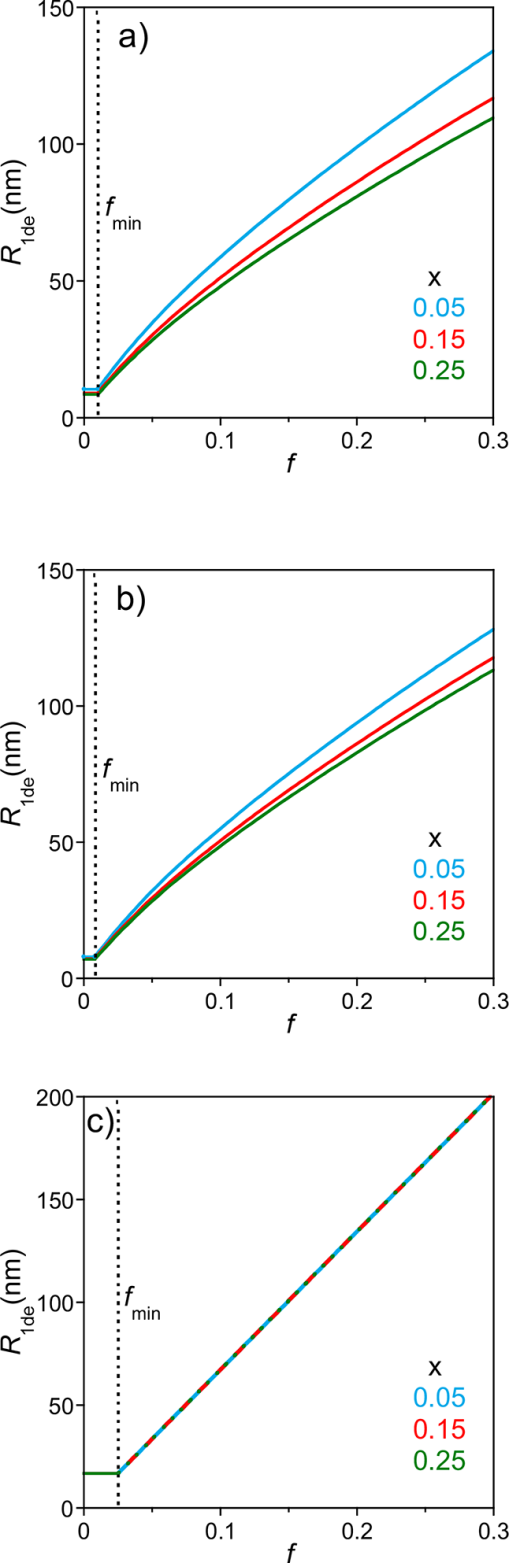
*R*_1de_ vs *f* for SCNPs with dipolar and electrostatic interactions in a good solvent at high dilution (*b* = 0.5 nm, *v*_d0_ = −2 nm^3^, *A* = 15 nm^–2^, *u* = 1.4, and *N* = 500) as a function of the intrachain cross-linking degree (*x*) for: (a) *s* = 0.15 (|*s* ^2^ *v*_d0_| ≪ *b* ^3^); (b) *s* = 0.25 (|*s* ^2^ *v*_d0_| = *b* ^3^); and (c) *s* = 0.3 (|*s* ^2^ *v*_d0_| ≫ *b* ^3^). Weak-PE behavior: *f* > *f*_min_ (see the text for details).

#### 2.4.2 SCNPs with Intrachain Covalent, Dipolar, and Electrostatic Interactions in a Good Solvent at High Dilution with Added Salt

We assume that similar to the case of the precursor chain (Section 2.3), above *c*_s_^*^ (eq 46), the SCNPs will become an SAW of electrostatic screening blobs with a size now given by^45^

$$R_{1\mathrm{de}}\approx bN^{3/5}(cb^{3}{)}^{-1/5}\Psi^{-2/5}{\left(1+\frac{2c_{s}}{fc}\right)}^{-1/5}$$ 60

where the specific values of Ψ are given by eqs 42, 54, and 59 for the cases of |*s* ^2^ *v*_d0_| *≫b* ^3^, |*s* ^2^ *v*_d0_| ≪ *b* ^3^, and |*s* ^2^ *v*_d0_| =*b* ^3^, respectively.

Figure 12 shows how the addition of salt affects the size and conformation of SCNPs of different cross-linking degrees and the balance between attractive dipolar interactions and excluded volume effects, as determined from eq 60.

**Figure 12:**
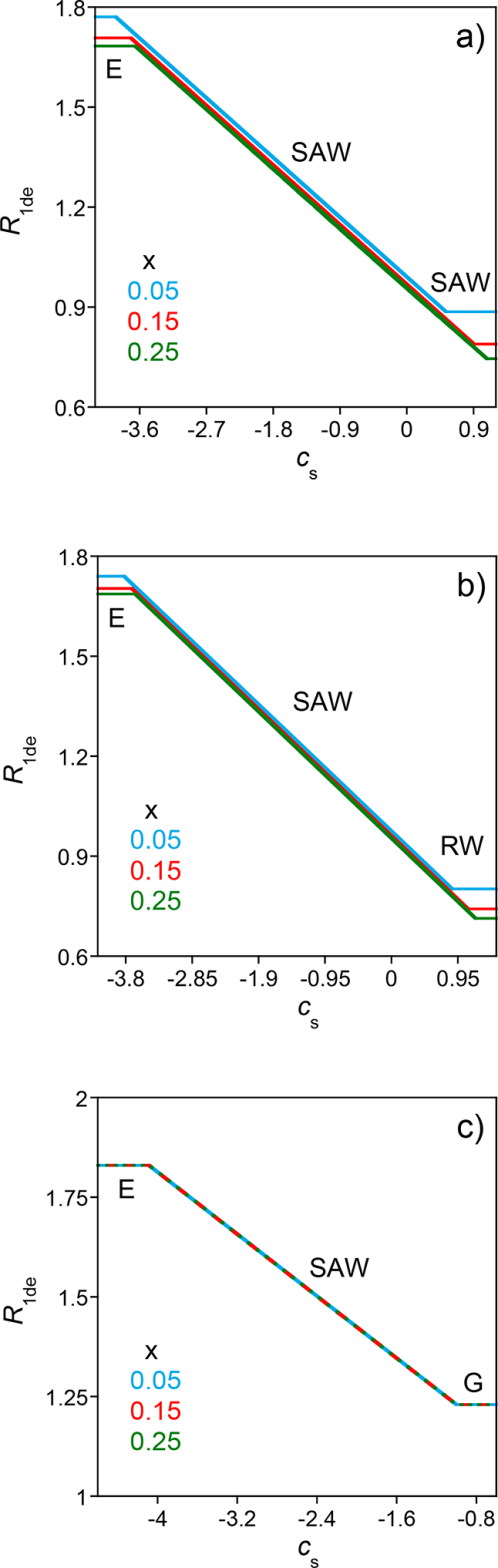
*R*_1de_ vs *c*_s_ (Log–Log plot) for SCNPs with dipolar and electrostatic interactions in a good solvent at high dilution (*b* = 0.5 nm, *v*_d0_ = −2 nm^3^, κ^–1^ (nm) $≅\frac{0.3}{\sqrt{c_{s}}},r_{0}$ = 0.25 nm, *A* = 15 mn^–2^, *u* = 1.4, *f* = 0.1, *N* = 500, and *c* = 10^–5^ M) as a function of the balance between attractive–dipolar– and repulsive–excluded volume–interactions and the intrachain cross-linking degree (*x*): (a) *s* = 0.15, |*s* ^2^ *v*_d0_| ≪ *b* ^3^; (b) *s* = 0.25, |*s* ^2^ *v*_d0_| = *b* ^3^; and (c) *s* = 0.3, |*s* ^2^ *v*_d0_| ≫ *b* ^3^. Conformation denoted as E, extended; SAW, self-avoiding walk; RW, random walk; and G, globular. Electrostatic interactions are only relevant for *c*_s_ < *c*_s_^*^ (see the text for details).

## 3 Discussion

### 3.1 SCNPs with Intramolecular Cross-Linking and Dipolar Interactions

First, we have generalized the standard ESN model^40,42–44^ to determine the size, conformation, and local domain size of SCNPs with intrachain covalent bonds (i.e., intramolecular cross-linking) and dipolar interactions, by combining the standard ESN model with the MFD theory of polyzwitterion solutions,^76^ as well as subsequent scaling analysis.^41^ We have considered the case of a very good solvent, high dilution (*c* < c*), and the presence (or absence) of added salt, the most relevant conditions to guide the experimental design of polyzwitterion-based SCNPs as artificial IDPs with potential application in, e.g., drug delivery and gene therapy.^50,51^ The case *c* > c* has been discussed in the SI.

In the case of globally neutral SCNPs with intrachain covalent and dipolar interactions, different scaling laws resulted depending on the balance between attractive dipolar interactions and repulsive excluded volume effects, as summarized in Table 3. First, if the attractive dipolar interactions are much weaker than the repulsive interactions (i.e., |*s* ^2^ *v*_d_| ≪ *b* ^3^), we find that the presence of dipolar interactions does not modify the scaling of the SCNP size on *N* (*R*_1d_ ∝ *N* ^2/5^) when compared to that of classical SCNPs without intrachain dipolar interactions,^40,42–44^ but does reduce its size. In general, we expect dipolar interactions to become relevant only if they are stronger than the thermal energy (i.e., $k_{B}T\left(|v_{d}|\frac{(sN{)}^{2}}{R_{1d}^{3}}\right)>k_{B}T$ ). Experimentally, the dipolar interactions can be tuned by several parameters: the fraction of zwitterionic monomers, *s*, the total number of monomers in the precursor chain, *N*, the strength of the dipolar interactions in the absence of added salt, *v*_d0_, which will depend on the chemical structure of the zwitterionic monomer, and the concentration of added monovalent salt, *c*_s_. Next, if the attractive forces balance with the repulsive ones (i.e., |*s* ^2^ *v*_d_| = *b* ^3^), then the SCNPs adopt the conformation of classical SCNPs in a theta solvent^40,42–44^ (*R*_1d_ ≈ *b* ^3/4^ *K* ^–1/8^ *N* ^3/8^) even being in a very good solvent (*v*_ev_ ≠ 0). We provide in Table 3 expressions of the local domain size and number of monomers per domain for SCNPs in which |*s* ^2^ *v*_d_| ≪ *b* ^3^ or |*s* ^2^ *v*_d_| = *b* ^3^. Finally, if the attractive dipole–dipole forces become much stronger (i.e., |*s* ^2^ *v*_d_| ≫ *b* ^3^), the conformation of the SCNPs becomes globular (*R*_0d_ ∝ *N* ^1/3^) and the solvent becomes a bad solvent for the SCNPs since *v*_eff_ = *b* ^3^ + *s* ^2^ *v*_d_ < 0. Interestingly, this model can be used to rationalize the collapsed conformations of SCNPs prepared by Qian and co-workers^102^ at an extremely low intrachain cross-linking degree (see the SI). Additionally, the model reproduces the scaling of the size with the intrachain cross-linking degree observed in coarse-grained MD simulations of globally neutral IDPs by Li and Hou, in which intramolecular cross-links are used as conformational regulators (see the SI).^103^

**Table 3:** Size, Conformation, Local Domain Size, and Number of Monomers per Domain of SCNPs with Covalent and Dipolar Interactions between Zwitterionic Monomers in a Very Good Solvent at High Dilution as a Function of the Balance between Dipolar Forces and Excluded Volume Interactions^a^

balance of interactions	size	conformation (scaling exponent, ν)
dipolar ≪ excluded volume |*s* ^2^ *v*_d_| ≪ *b* ^3^	$R_{1d}\approx b^{3/5}{\left(1+\frac{s^{2}v_{d}}{b^{3}}\right)}^{1/5}K^{-1/5}N^{2/5}$	collapsed (ν = 2/5)
dipolar = excluded volume |*s* ^2^ *v*_d_| = *b* ^3^	$R_{1d}\approx b^{3/4}K^{-1/8}N^{3/8}$	collapsed (ν = 3/8)
dipolar ≫ excluded volume |*s* ^2^ *v*_d_| ≫ *b* ^3^	$R_{1d}\approx b{\left(\left|1+\frac{s^{2}v_{d}}{b^{3}}\right|\right)}^{-1/3}N^{1/3}$	globular (ν = 1/3)

balance of interactions	local domain size	number of monomers per domain
dipolar ≪ excluded volume |*s* ^2^ *v*_d_| ≪ *b* ^3^	$$\xi_{d}\approx R_{1d}{\left(\frac{R_{1d}}{R_{0d}}\right)}^{5/3}$$	$g_{d}\approx N{\left(\frac{R_{1d}}{R_{0d}}\right)}^{5}$
dipolar = excluded volume |*s* ^2^ *v*_d_| = *b* ^3^	$$\xi_{d}\approx R_{1d}{\left(\frac{R_{1d}}{R_{0d}}\right)}^{8/3}$$	$g_{d}\approx N{\left(\frac{R_{1d}}{R_{0d}}\right)}^{8}$
dipolar ≫ excluded volume^b^ |*s* ^2^ *v*_d_| ≫ *b* ^3^		

a *R*_1d_, size of SCNPs with intrachain covalent and dipolar interactions (*R*_1d_ ∝ *N* ^v^); *b,* monomer size; *s,* fraction of zwitterionic monomers; *v*_d_= *v*_d0_(1 + κ*r*_0_)­e^–κ*r*_0_^, where *v*_d0_ is the strength of the dipolar interactions in the absence of added salt, κ is the inverse Debye length, and *r*_0_ is the separation distance between dipoles; *K =* *Ax,* where *A* is the elasticity constant of the SCNP and *x* is the fraction of intrachain cross-linked monomers; *N,* total number of monomers; ξ_d_, size of the local domains in the SCNPs; *R*_0d_, size of the precursor chain; and *g*_d_, number of monomers per local domain (see Section 2.2 for details).

b The scaling approach followed to obtain the expressions for ξ_d_ and *g*_d_ is not valid in this case.

Figure 13 illustrates the values of the scaling exponent for *N* corresponding to the precursor chain and SCNPs as a function of the balance between dipolar and excluded volume interactions. Of special mention is the fact that SCNPs prepared from precursors under the |*s* ^2^ *v*_d_| ≫ *b* ^3^ conditions are predicted to retain their size even if intramolecularly cross-linked (see Section 2.2). According to the model, SCNPs with intrachain covalent and dipolar interactions can also show the anti-polyelectrolyte effect,^48^ as previously illustrated in Figure 6. This effect offers an additional parameter to tune the size and even the conformation of SCNPs with intrachain covalent and dipolar interactions when compared to classical SCNPs.

**Figure 13:**
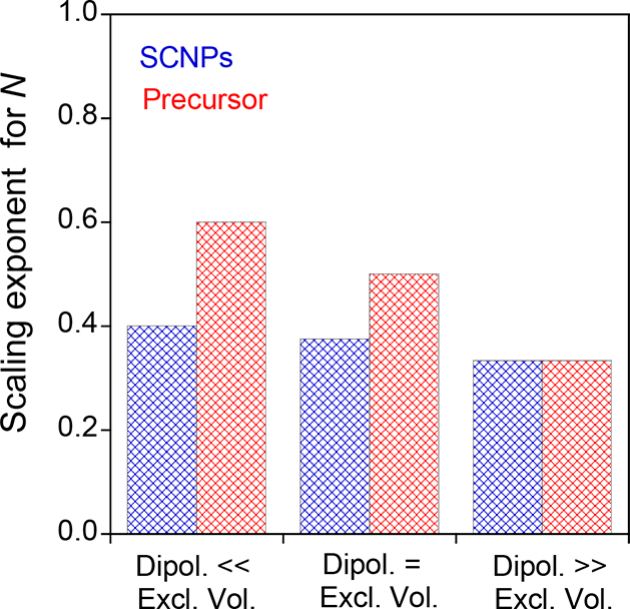
Comparison of the scaling exponent for *N* of globally neutral SCNPs with intrachain covalent and dipolar interactions (blue grid bars) vs the corresponding precursor chain (red grid bars), both in a very good solvent at high dilution, as a function of the balance between dipolar and excluded volume interactions (see Table 3).

### 3.2 SCNPs with Covalent, Dipolar, and Electrostatic Interactions

For SCNPs with intrachain covalent, dipolar, and electrostatic interactions, Table 4 gives a summary of the size and conformations of these SCNPs in a very good solvent at high dilution (without added salt) and semidiluted conditions (see the SI) as a function of the balance between dipolar forces and excluded volume interactions. In general, the presence of repulsive, long-range electrostatic interactions above a certain threshold, *f*_min_, has a profound effect on the size and conformation of these SCNPs when compared to SCNPs with only intrachain covalent and dipolar interactions. In the absence of added salt, for *f* < *f*_min_, the size and conformation are those provided in Table 3. However, for *f* > *f*_min_, the SCNPs at high dilution (*c* < c*) adopt extended conformations (*R*_1de_ ∝ *N*) in all of the cases, as indicated in Table 4. In fact, intrachain cross-linking of SCNPs with intrachain covalent, dipolar, and electrostatic interactions at *f* > *f*_min_ is only possible inside the corresponding electrostatic blobs (i.e., local domains), which repel each other to give the overall extended configuration of the SCNPs. The model provides useful expressions to estimate the size of the cross-linked electrostatic blobs, *D*_c_, and the number of monomers inside each blob, *g*_c_ (see Table 4). Once again, SCNPs prepared from precursors under the |*s* ^2^ *v*_d_| ≫ *b* ^3^ conditions are predicted to retain the size of the precursor even if intramolecularly cross-linked. Screening of the repulsive electrostatic interactions upon addition of a salt above a critical value, *c*_s_^*^, has a profound effect on the size of these SCNPs that follow the conventional PE behavior^45,46^ in a large range of salt concentrations (see Figure 12).

**Table 4:** Local Domain Size, Number of Monomers per Domain, Size, and Conformation of SCNPs with Intrachain Covalent, Dipolar, and Electrostatic Interactions in a Very Good Solvent (No Added Salt) as a Function of the Balance between Dipolar Forces and Excluded Volume Interactions^a^

balance of interactions	local domain size	number of monomers per domain
highly diluted conditions (c < c*) and semidiluted conditions (c* < c < c**)		
dipolar ≪ excluded volume |*s* ^2^ *v*_d0_| ≪ *b* ^3^	$D_{c}\approx b^{1/2}{\left(1+\frac{s^{2}v_{d0}}{b^{3}}\right)}^{1/4}K^{-1/4}u^{-1/4}f^{-1/2}$	$$g_{c}\approx b^{-1/4}{\left(1+\frac{s^{2}v_{d0}}{b^{3}}\right)}^{1/8}K^{-1/8}u^{-5/8}f^{-5/4}$$
dipolar = excluded volume |*s* ^2^ *v*_d0_| = *b* ^3^	$D_{c}\approx b^{9/13}K^{-2/13}u^{-3/13}f^{-6/13}$	$g_{c}\approx b^{-2/13}K^{-1/13}u^{-8/13}f^{-16/13}$
dipolar ≫ excluded volume |*s* ^2^ *v*_d0_| ≫ *b* ^3^	$D_{c}\approx bu^{-1/3}f^{-2/3}$	$g_{c}\approx |1+\frac{s^{2}v_{d0}}{b^{3}}|u^{-1}f^{-2}$

interactions balance	size^b^	conformation (scaling exponent, ν)
highly diluted conditions (c < c*)		
dipolar ≪ excluded volume |*s* ^2^ *v*_d0_| ≪ *b* ^3^	$R_{1\mathrm{de}}\approx b^{3/4}{\left(1+\frac{s^{2}v_{d0}}{b^{3}}\right)}^{1/8}K^{-1/8}Nu^{3/8}f^{3/4}$	extended (ν = 1)
dipolar = excluded volume |*s* ^2^ *v*_d0_| = *b* ^3^	$R_{1\mathrm{de}}\approx b^{11/13}K^{-1/13}Nu^{5/13}f^{10/13}$	extended (ν = 1)
dipolar ≫ excluded volume |*s* ^2^ *v*_d0_| ≫ *b* ^3^	$R_{1\mathrm{de}}\approx b{\left(\left|1+\frac{s^{2}v_{d0}}{b^{3}}\right|\right)}^{-1/2}Nu^{1/2}f$	extended^c^ (ν = 1)
semidiluted conditions (c* < c < c**)		
dipolar ≪ excluded volume |*s* ^2^ *v*_d0_| ≪ *b* ^3^	$R_{c1\mathrm{de}}\approx b^{3/16}{\left(1+\frac{s^{2}v_{d0}}{b^{3}}\right)}^{1/32}K^{-1/32}N^{1/2}u^{3/32}f^{3/16}c^{-1/4}$	random walk (ν = 1/2)
dipolar = excluded volume |*s* ^2^ *v*_d0_| = *b* ^3^	$R_{c1\mathrm{de}}\approx b^{11/52}K^{-1/52}N^{1/2}u^{5/52}f^{5/26}c^{-1/4}$	random walk (ν = 1/2)
dipolar ≫ excluded volume |*s* ^2^ *v*_d0_| ≫ *b* ^3^	$R_{c1\mathrm{de}}\approx b^{1/4}{\left(\left|1+\frac{s^{2}v_{d0}}{b^{3}}\right|\right)}^{-1/8}N^{1/2}u^{1/8}f^{1/4}c^{-1/4}$	random walk^c^ (ν = 1/2)

a $c^{*}=\frac{N}{R_{1\mathrm{de}}^{3}}$ ; $c^{**}=c^{*}{\left(\frac{R_{1\mathrm{de}}}{D_{c}}\right)}^{2}$ ; *D*_c_, size of the cross-linked electrostatic blob; *g*_c_, number of monomers per cross-linked electrostatic blob; *b,* monomer size; *s,* fraction of zwitterionic monomers; *v*_d0_, strength of the dipolar interactions in the absence of added salt; *u* = *l*_B_/*b* (*l*_B_ is the Bjerrum length); *f,* fraction of charged monomers; *K* = *Ax,* where *A* is the elasticity constant of the SCNP and *x* is the fraction of intrachain cross-linked monomers; *N,* total number of monomers; *R*_1de_, size of the SCNPs with intrachain dipolar and electrostatic interactions (*R*_1de_ ∝ *N* ^v^) at *c* < c*; and *R*_c1de_, size of the SCNPs with intrachain dipolar and electrostatic interactions at c*< *c* < c**.

b *f*_min_ < *f* < *f*_max_ (see Section 2.3 for details).

c Necklace globule conformation.

Figure 14 shows the values of the scaling exponent on *f* of the size of the SCNPs and the corresponding precursor chain as a function of the balance between dipolar and excluded volume interactions. On the one hand, at high dilution (Figure 14a), a clear increase in the scaling exponent on *f* is observed for SCNPs in which |*s* ^2^ *v*_d_| ≪ *b* ^3^ or |*s* ^2^ *v*_d_| = *b* ^3^ when compared to its respective precursors (31 and 15%, respectively). Under semidilute conditions (Figure 14b), the scaling exponent of *f* undergoes a 4-fold reduction in all cases with respect to the values at high dilution conditions, and the SCNPs adopt an RW conformation (ν = 1/2).

**Figure 14:**
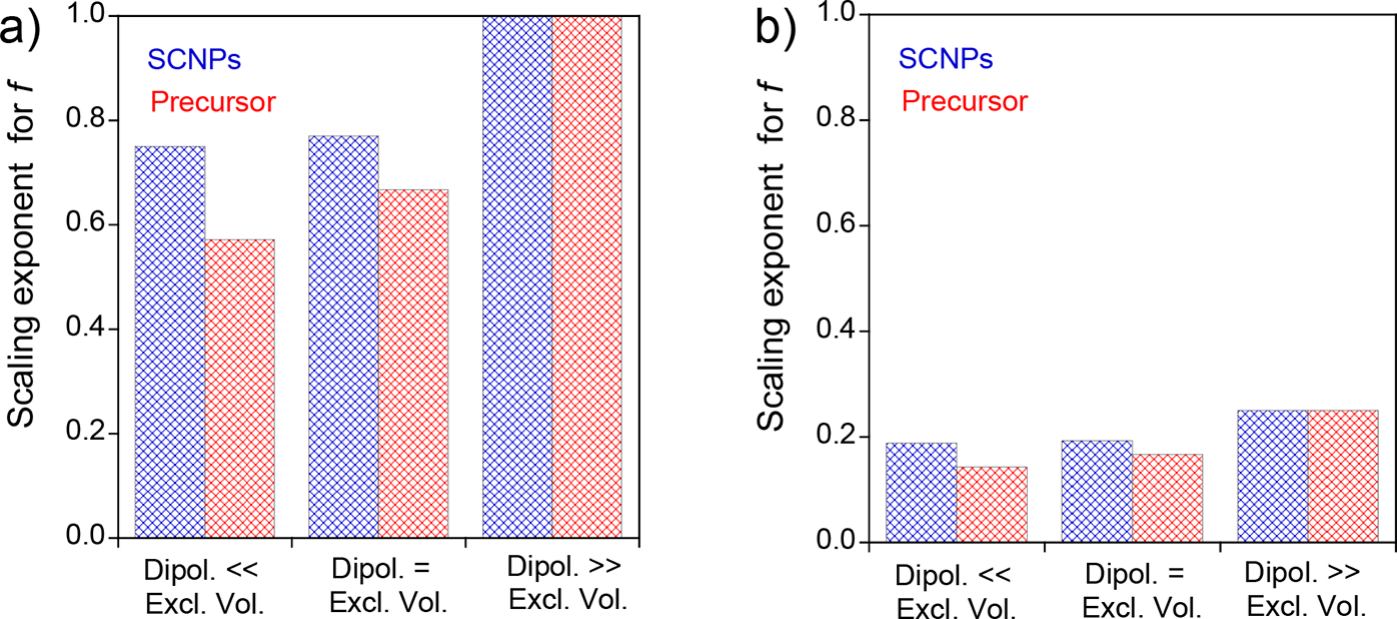
Comparison of the scaling exponent for *f* of SCNPs with intrachain covalent, dipolar, and electrostatic interactions (blue grid bars) vs the corresponding precursor chain (red grid bars), both in a very good solvent at high dilution, under (a) highly diluted (*c* < c*) and (b) semidiluted (c* < *c* < c**) conditions, a function of the balance between dipolar and excluded volume interactions (see Table 4).

### 3.3 Design Principles toward Artificial IDPs based on SCNPs with Covalent, Dipolar, and Electrostatic Interactions

IDPs are prevalent and play a crucial role in cellular signaling. The analogy between SCNPs and IDPs concerning conformational heterogeneity (topology dispersity) and the presence of locally compact domains was previously pointed out by some of us, suggesting that concentrated solutions of SCNPs can be used as a simple model for IDPs under crowding conditions.^82,104^ Recently, Li and Hou^103^ investigated, through coarse-grained MD simulations, the intrachain cross-linking of model IDPs and concluded that cross-link-induced conformation changes have a nontrivial effect on the phase separation dynamics and thermodynamics of IDPs.

Figure 15, together with Tables 3 and 4, is extremely useful to guide the design of artificial IDPs based on SCNPs with covalent, dipolar, and electrostatic interactions. In particular, Figure 15 illustrates how the conformation of artificial IDPs based on SCNPs having covalent, dipolar, and electrostatic interaction can be tuned depending on the fraction of monomers per SCNP carrying the elemental electrical charge, *f*, and the SCNP concentration in solution, *c*, as a function of the balance between attractive dipolar and repulsive excluded volume forces. Additional structural parameters to tune the size of these artificial IDPs are the degree of cross-linking, *x* (only if dipolar interactions are not stronger than the excluded volume forces), the fraction of zwitterionic monomers, *s*, and the total number of monomers per SCNP, *N*.

**Figure 15:**
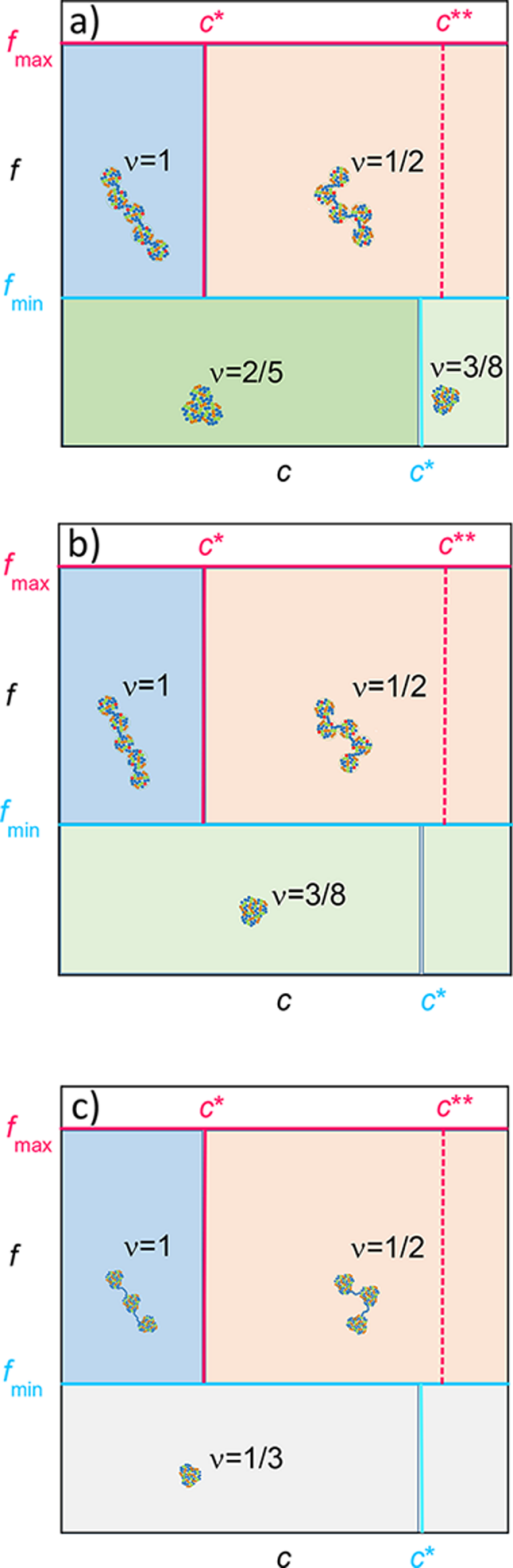
Conformational diagram (*R* ∝ *N* ^v^) in *f* vs. *c* coordinates (Log–Log plot) corresponding to SCNPs with intrachain covalent, dipolar, and electrostatic interactions in a good solvent for (a) attractive dipolar interactions much weaker than the repulsive excluded volume interactions, (b) attractive dipolar interactions balanced with repulsive excluded volume interactions, and (c) attractive dipolar interactions stronger than repulsive excluded volume interactions. c* and c** are the chain and electrostatic blob overlap concentrations, respectively (see the text for details).

#### 3.3.1 Artificial IDPs with Expanded Conformation

Around 66% of the intrinsically disordered human proteome (28058 IDRs) recently analyzed by Tesei et al.^75^ showed conformational ensembles that approximate the ideal RW configuration (ν ≈ 1/2), whereas 32% adopted more extended conformations (ν > 0.55).

Let us assume that we want to construct artificial IDPs with an expanded conformation under good solvent conditions (water). According to Figure 15a, by selecting a water-soluble precursor with a fraction of monomers per SCNP carrying the elemental electrical charge higher than *f*_min_ (see eq 33), we enter the region of extended conformations (upper part of Figure 15a). Since the c* of SCNPs with *f* > *f*_min_ is often extremely low (orders of magnitude lower than c* for globally neutral SCNPs), we expect SCNPs from this precursor to show an RW configuration (ν ≈ 1/2) at typical experimental conditions (*c* ≫ c*). Notice that if enough monovalent salt is present (i.e., *c*_s_ > *c*_s_*), we expect the artificial IDP to reach the SAW regime (ν ≈ 3/5). In this regard, it should be noted that the physiological conditions correspond to 0.15 M monovalent salt.

#### 3.3.2 Artificial IDPs with Compact Conformation

Perhaps most interesting is the construction of artificial IDPs with a compact conformation under good solvent conditions. Only 2% of the intrinsically disordered human proteome recently investigated comprises substantially compact IDRs with ν < 0.45, some of them related to the formation of biomolecular condensates and intracellular membraneless organelles.^75^ Within cells, biomolecular condensates form dynamic, membraneless domains, whose functions remain partly elusive. IDPs commonly act as structural frameworks, promoting their assembly via liquid–liquid phase separation (LLPS).^105^

According to Figure 15, to design artificial IDPs with a compact conformation in a good solvent based on SCNPs having covalent, dipolar, and electrostatic interactions, it is necessary to work with a precursor with a fraction of monomers per SCNP carrying the elemental electrical charge lower than *f*_min_ to enter the region of collapsed conformations (lower part of Figure 15a–c). At *c* < c*, the size scaling exponent will depend on the balance between dipolar and excluded volume interactions, decreasing from ν ≈ 2/5 to ν ≈ 3/8 and ν ≈ 1/3 upon increasing the strength of the dipolar attractive forces. However, as discussed previously, if dipolar interactions are stronger than the excluded volume forces (ν ≈ 1/3), then the solvent becomes a bad solvent, so in real experiments, multichain aggregation phenomena and even macroscopic precipitation of the SCNPs out of the solution are expected. Consequently, to avoid aggregation issues, it would be preferable to design artificial IDPs with dipolar interactions weaker than excluded volume forces (ν ≈ 2/5). For this case, as illustrated in Figure 15a, the size scaling exponent is expected to decrease from ν ≈ 2/5 to ν ≈ 3/8 at a high concentration of SCNPs in solution (*c* > c*). Contrary to the case of artificial IDPs with *f* > *f*_min_, we do not expect the size scaling exponent to change in the presence of a monovalent salt (see Table 3). We surmise artificial IDPs with dipolar interactions stronger than excluded volume forces (ν ≈ 1/3) could be a good model system to investigate LLPS phenomena.

## 4 Conclusions

In summary, we have developed a useful model to guide the rational design of ionic SCNPs with intrachain covalent bonds (i.e., intramolecular cross-links), dipolar, and electrostatic interactions, as simplified analogues of natural IDPs. At the scaling level of accuracy, this work unravels the effect of multiple interactions (from short- to long-range forces) on the total and local size as well as configuration of these SCNPs. The size and conformation of these complex SCNPs depends on a variety of parameters including the fraction of reactive, dipolar, and electrostatic monomers in the precursor, the total number of monomers and the monomer length, the strength of the dipole–dipole interactions, the elasticity constant of the SCNPs, the Bjerrum length, the concentration of added salt, the degree of dilution of the solution, and the solvent quality. We have derived useful expressions to estimate the size and number of local compact domains in these complex SCNPs. Based on this theoretical work, we envision the practical design of artificial IDPs based on tunable multiresponsive polyzwitterionic SCNPs as advanced nanomaterials of great interest for applications in nanomedicine, the development of antifreeze and antifouling coatings, hydrogel materials, etc.

## Supplementary Material



## Notes

Semidiluted solutions of SCNPs with intrachain covalent and dipolar interactions; semidiluted solutions of a linear precursor of SCNPs with intrachain covalent, dipolar, and electrostatic interactions; semidiluted solutions of SCNPs with intrachain covalent, dipolar, and electrostatic interactions; collapsed conformations of SCNPs prepared at extremely low intrachain cross-linking degree; and scaling of the size with the intrachain cross-linking degree observed in coarse-grained MD simulations of globally neutral IDPs (PDF)

## Nomenclature

*A*: elasticity constant of single-chain nanoparticles
*b*: monomer size
*c*: polymer (or single-chain nanoparticle) concentration in solution
*c**: chain overlap concentration (*c**= *N*/*R* ^3^)
*c***: electrostatic blob overlap concentration (*c*** = *c**­[*R*/*D*]^2^)
*c*_s_: concentration of salt added in solution
*c*_s_*: critical concentration of added salt in solution below which electrostatic interactions are relevant
*D*: electrostatic blob size (diameter)
*D*_e_: electrostatic blob size of a precursor chain with reactive, zwitterionic, charged, and inert monomers
*D*_c_: cross-linked electrostatic blob size (i.e., local domain size) of a single-chain nanoparticle with intrachain covalent, dipolar, and electrostatic interactions
*e*: elementary charge
*f*: fraction of monomers carrying the elemental electrical charge
*f*_min_: minimum fraction of charged monomers for electrostatic interactions to be relevant
*f*_max_: maximum fraction of charged monomers for the model to be valid
*f*_c_: critical charge fraction between states of the necklace globule with different numbers of beads
*F*: free energy of an isolated precursor chain (or a single-chain nanoparticle) in solution
*F*_1_: contribution to *F* from entropic elasticity
*F*_2_: contribution to *F* from binary excluded volume interactions
*F*_3_: contribution to *F* from ternary excluded volume interactions
*F*_4_: contribution to *F* from dipole–dipole interactions
*g*_c_: number of monomers in a cross-linked electrostatic blob of a single-chain nanoparticle with intrachain covalent, dipolar, and electrostatic interactions
*g*_d_: number of monomers per local domain in single-chain nanoparticles with intrachain and dipolar interactions
*g*_e_: number of monomers in an electrostatic blob of a precursor chain with reactive, zwitterionic, charged, and inert monomers
*k*_B_: Boltzmann constant
*K*: elasticity parameter of single-chain nanoparticles (*K* = *Ax*)
*l*_B_: Bjerrum length ( $l_{B}=\frac{e^{2}}{4\pi \varepsilon k_{B}T}$ )
*l*_p_: electrostatic persistence length
*N*: total number of monomers in a precursor chain (or single-chain nanoparticle)
*N*_bead_: number of beads of the necklace globule (*N*_bead_ = 1, 2, 3, ···)
*p*_0_: dipole length
*r*_0_: separation distance between dipoles in a quadrupole
*R*: size (end-to-end distance)
*R*_0_: size of a precursor chain with only reactive and inert monomers (*c* < *c**)
*R*_0d_: size of a precursor chain with reactive, zwitterionic, and inert monomers (*c* < *c**)
*R*_1d_: size of a single-chain nanoparticle with intrachain covalent and dipolar interactions (*c* < *c**)
*R*_0de_: size of a precursor chain with reactive, zwitterionic, charged, and inert monomers (*c* < *c**)
*R*_1de_: size of a single-chain nanoparticle with intrachain covalent, dipolar, and electrostatic interactions (*c* < *c**)
*R*_c0de_: size of a precursor chain with reactive, zwitterionic, charged, and inert monomers (*c** < *c* < *c***)
*R*_c1de_: size of a single-chain nanoparticle with intrachain covalent, dipolar, and electrostatic interactions (*c** < *c* < *c***)
*s*: fraction of zwitterionic monomers
*s*_max_: critical fraction of zwitterionic monomers that renders the precursor chain insoluble in the solvent
*T*: temperature
*u*: ratio of Bjerrum length to monomer size (*u* = *l*_B_/*b*)
*v*_d_: dipole–dipole interaction parameter (*v*_d_ = *v*_d0_(1 + κ*r*_0_)­e^–κ*r*_0_^, quenched quadrupoles)
*v*_d0_: dipole–dipole interaction parameter in the absence of added salt
*v*_eff_: effective binary excluded volume parameter in the presence of dipolar interactions (*v*_eff_ ≡ *v*_ev_ + *g* ^2^ *v*_d0_)
*v*_ev_: binary excluded volume parameter (*v*_ev_ = *b* ^3^ in a very good, athermal solvent)
*w*: ternary excluded volume parameter (*w* = *b* ^6^ in a very good, athermal solvent)
*x*: fraction of reactive monomers (also intrachain cross-linking degree in SCNPs)
β: inverse of thermal energy (β^–1^ ≡ *k*_B_ *T*)
δ: scaling exponent of *R*_1d_ on *x*
ε: dielectric constant of solvent
κ: inverse Debye length (κ^–1^ (nm) $≅0.3/\sqrt{c_{s}}$ , monovalent salts in water)
ν: scaling exponent of *R* on *N*
τ: effective value of the reduced temperature parameter ( $\tau \approx |\frac{v_{\mathrm{eff}}}{b^{3}}|$ , *v*_eff_ < 0)
ξ_d_: size of local domains in single-chain nanoparticles with intrachain covalent and dipolar interactions
ξ_T_: thermal blob size ( $\xi_{T}\approx \frac{b^{4}}{\left|v_{\mathrm{eff}}\right|}$ )
Ψ: stretching parameter (Ψ = *bN*/*R*)

## References

[1] Single-chain polymer nanoparticles: synthesis, characterization, and applications, Pomposo, J. A. , Ed.; Wiley-VCH, 2017.

[2] Arena D., Verde-Sesto E., Rivilla I., Pomposo J. A. (2024). Artificial Photosynthases:
Single-Chain Nanoparticles with Manifold Visible-Light Photocatalytic
Activity for Challenging ″in Water″ Organic Reactions. J. Am. Chem. Soc..

[3] Mundsinger K., Izuagbe A., Tuten B. T., Roesky P. W., Barner-Kowollik C. (2024). Single chain
nanoparticles in catalysis. Angew. Chem., Int.
Ed..

[4] Liu Y. L., Pujals S., Stals P. J. M., Paulöhrl T., Presolski S. I., Meijer E. W., Albertazzi L., Palmans A. R. A. (2018). Catalytically Active Single-Chain Polymeric Nanoparticles:
Exploring Their Functions in Complex Biological Media. J. Am. Chem. Soc..

[5] Rothfuss H., Knöfel N. D., Roesky P. W., Barner-Kowollik C. (2018). Single-chain
nanoparticles as catalytic nanoreactors. J.
Am. Chem. Soc..

[6] Rubio-Cervilla J., González E., Pomposo J. A. (2017). Advances in single-chain nanoparticles
for catalysis applications. Nanomaterials.

[7] Pinacho-Olaciregui J., Verde-Sesto E., Taton D., Pomposo J. A. (2024). Lanthanide-based
single-chain nanoparticles as “visual” pass/fail sensors
of maximum permissible concentration of Cu^2+^ ions in drinking
water. Macromol. Rapid Commun..

[8] Zeng Y. M., Xu T. C., Chen W. Z., Fang J. L., Chen D. Z. (2024). Quasi-Chromophores
Segregated by Single-Chain Nanoparticles of Fluorinated Zwitterionic
Random Copolymers Showing Remarkably Enhanced Fluorescence Emission
Capable of Fluorescent Cell Imaging. Macromol.
Rapid Commun..

[9] Maag P. H., Feist F., Frisch H., Roesky P. W., Barner-Kowollik C. (2024). Förster
resonance energy transfer within single chain nanoparticles. Chem. Sci..

[10] Lu Z. M., Zhang J. Y., Yin W., Guo C. F., Lang M. D. (2022). Preparation
of AIE Functional Single-Chain Polymer Nanoparticles and Their Application
in H_2_O_2_ Detection through Intermolecular Heavy-Atom
Effect. Macromol. Rapid Commun..

[11] De-La-Cuesta J., Verde-Sesto E., Arbe A., Pomposo J. A. (2021). Self-Reporting of
Folding and Aggregation by Orthogonal Hantzsch Luminophores Within
a Single Polymer Chain. Angew. Chem., Int. Ed..

[12] Arena D., Nguyen C., Ali L. M. A., Verde-Sesto E., Iturrospe A., Arbe A., Isci U., Sahin Z., Dumoulin F., Gary-Bobo M., Pomposo J. A. (2024). Amphiphilic Single-Chain
Polymer Nanoparticles as Imaging and Far-Red Photokilling Agents for
Photodynamic Therapy in Zebrafish Embryo Xenografts. Adv. Healthcare Mater..

[13] Vo Y., Nothling M. D., Raveendran R., Cao C., Stenzel M. H. (2024). Effects
of Drug Conjugation on the Biological Activity of Single-Chain Nanoparticles. Biomacromolecules.

[14] Hamelmann N. M., Paulusse J. M. J. (2023). Single-chain polymer nanoparticles
in biomedical applications. J. Controlled Release.

[15] Kröger A. P. P., Paulusse J. M. J. (2018). Single-chain
polymer nanoparticles
in controlled drug delivery and targeted imaging. J. Controlled Release.

[16] Cheng C. C., Lee D. J., Liao Z. S., Huang J. J. (2016). Stimuli-responsive
single-chain polymeric nanoparticles towards the development of efficient
drug delivery systems. Polym. Chem..

[17] Wijker S., Palmans A. R. A. (2023). Protein-Inspired
Control over Synthetic Polymer Folding
for Structured Functional Nanoparticles in Water. ChemPlusChem.

[18] Nitti A., Carfora R., Assanelli G., Notari M., Pasini D. (2022). Single-Chain
Polymer Nanoparticles for Addressing Morphologies and Functions at
the Nanoscale: A Review. ACS Appl. Nano Mater..

[19] Verde-Sesto E., Arbe A., Moreno A. J., Cangialosi D., Alegría A., Colmenero J., Pomposo J. A. (2020). Single-chain nanoparticles:
opportunities provided by internal and external confinement. Mater. Horiz..

[20] Huurne G. M., Palmans A. R. A., Meijer E. W. (2019). Supramolecular Single-Chain Polymeric
Nanoparticles. CCS Chem..

[21] Shao Y., Yang Z. Z. (2022). Progress in polymer single-chain
based hybrid nanoparticles. Prog. Polym. Sci..

[22] Alqarni M. A. M., Waldron C., Yilmaz G., Becer C. R. (2021). Synthetic Routes
to Single Chain Polymer Nanoparticles (SCNPs): Current Status and
Perspectives. Macromol. Rapid Commun..

[23] Chen J. F., Garcia E. S., Zimmerman S. C. (2020). Intramolecularly
Cross-Linked Polymers:
From Structure to Function with Applications as Artificial Antibodies
and Artificial Enzymes. Acc. Chem. Res..

[24] Mavila S., Eivgi O., Berkovich I., Lemcoff N. G. (2016). Intramolecular Cross-Linking
Methodologies for the Synthesis of Polymer Nanoparticles. Chem. Rev..

[25] Altintas O., Barner-Kowollik C. (2016). Single-Chain Folding of Synthetic
Polymers: A Critical
Update. Macromol. Rapid Commun..

[26] Gonzalez-Burgos M., Latorre-Sanchez A., Pomposo J. A. (2015). Advances in single chain technology. Chem. Soc. Rev..

[27] Lyon C. K., Prasher A., Hanlon A. M., Tuten B. T., Tooley C. A., Frank P. G., Berda P. G. (2015). A brief user’s guide to single-chain
nanoparticles. Polym. Chem..

[28] Sanchez-Sanchez A., Pérez-Baena I., Pomposo J. A. (2013). Advances in Click Chemistry for Single-Chain
Nanoparticle Construction. Molecules.

[29] Harth E., Horn B. V., Lee V. Y., Germack D. S., Gonzales C. P., Miller R. D., Hawker C. J. (2002). A facile
approach to architecturally
defined nanoparticles via intramolecular chain collapse. J. Am. Chem. Soc..

[30] Pomposo J. A. (2014). Bioinspired
single-chain polymer nanoparticles. Polym. Int..

[31] Latorre-Sánchez A., Pomposo J. A. (2016). Recent
bioinspired applications of single-chain nanoparticles. Polym. Int..

[32] Thümmler J. F., Binder W. H. (2024). Compartmentalised single-chain nanoparticles and their
function. Chem. Commun..

[33] Newberry R. W., Raines R. T. (2019). Secondary Forces
in Protein Folding. ACS Chem. Biol..

[34] Blazquez-Martín A., Verde-Sesto E., Moreno A. J., Arbe A., Colmenero J., Pomposo J. A. (2021). Advances in the Multi-Orthogonal Folding of Single
Polymer Chains into Single-Chain Nanoparticles. Polymers.

[35] Lo
Verso F., Pomposo J. A., Colmenero J., Moreno A. J. (2014). Multi-orthogonal folding of single polymer chains into
soft nanoparticles. Soft Matter.

[36] Han Z. X., Hilburg S. L., Alexander-Katz A. (2022). Forced Unfolding
of Protein-Inspired
Single-Chain Random Heteropolymers. Macromolecules.

[37] Martin J. E., Eichinger B. E. (1983). Dimensions
of intramolecularly crosslinked polymers.
1. Theory. Macromolecules.

[38] Martin J. E., Eichinger B. E. (1983). Dimensions
of intramolecularly crosslinked polymers.
2. Dilute solution thermodynamic parameters and photon correlation
results on the polystyrene/cyclopentane system. Macromolecules.

[39] Rabbel H., Breier P., Sommer J. U. (2017). Swelling Behavior of Single-Chain
Polymer Nanoparticles: Theory and Simulation. Macromolecules.

[40] De-La-Cuesta J., González E., Moreno A. J., Arbe A., Colmenero J., Pomposo J. A. (2017). Size of Elastic Single-Chain Nanoparticles
in Solution
and on Surfaces. Macromolecules.

[41] Pomposo J. A., Moreno A. J., Arbe A., Colmenero J. (2018). Local Domain
Size in Single-Chain Polymer Nanoparticles. ACS Omega.

[42] Pomposo J. A., Rubio-Cervilla J., Gonzalez E., Moreno A. J., Arbe A., Colmenero J. (2018). Ultrafiltration of single-chain polymer nanoparticles
through nanopores and nanoslits. Polymer.

[43] Asenjo
Sanz I., Moreno A. J., Arbe A., Colmenero J., Pomposo J. A. (2019). Brushes of Elastic Single-Chain Nanoparticles on Flat
Surfaces. Polymer.

[44] Arena D., Verde-Sesto E., Pomposo J. A. (2022). Stars, combs and bottlebrushes of
elastic single-chain nanoparticles. Polymer.

[45] Dobrynin A. V., Colby R. H., Rubinstein M. (1995). Scaling Theory
of Polyelectrolyte
Solutions. Macromolecules.

[46] Dobrynin A. V., Rubinstein M. (2005). Theory of
polyelectrolytes in solutions and at surfaces. Prog. Polym. Sci..

[47] Pomposo J. A., Arena D., Verde-Sesto E., Maiz J., de Molina P. M., Moreno A. J. (2024). Why Single-Chain
Nanoparticles from Weak Polyelectrolytes
Can Be Synthesized at Large Scale in Concentrated Solution?. Macromol. Rapid Commun..

[48] Lowe A. B., McCormick C. L. (2002). Synthesis
and solution properties of zwitterionic polymers. Chem. Rev..

[49] Ladenheim H., Morawetz H. (1957). A new type
of polyampholyte: Poly­(4-vinyl pyridine
betaine). J. Polym. Sci..

[50] Blackman L. D., Gunatillake P. A., Cass P., Locock K. E. S. (2019). An introduction
to zwitterionic polymer behavior and applications in solution and
at surfaces. Chem. Soc. Rev..

[51] Zheng L. C., Sundaram H. S., Wei Z. Y., Li C. C., Yuan Z. F. (2017). Applications
of zwitterionic polymers. React. Funct. Polym..

[52] Zeng Y. M., Xu T. C., Hou X. F., Liu J., Liu C. Q., Chang Z. S., Fang J. L., Chen D. Z. (2023). Enzyme
Stabilization
and Catalytic Activity Enhancement by Single-Chain Nanoparticles of
Fluorinated Zwitterionic Random Copolymers. ACS Appl. Polym. Mater..

[53] Dobrynin A., Rubinstein M. (1995). Flory Theory of a Polyampholyte Chain. J. Phys..

[54] Higgs P. G., Joanny J. F. (1991). Theory of polyampholyte solutions. J. Chem. Phys..

[55] Rumyantsev A. M., Jackson N. E., Johner A., de Pablo J. J. (2021). Scaling Theory of
Neutral Sequence-Specific Polyampholytes. Macromolecules.

[56] Kantor Y., Li H., Kardar M. (1992). Conformations
of Polyampholytes. Phys. Rev. Lett..

[57] Muthukumar M. (1996). Localized
structures of polymers with long-range interactions. J. Chem. Phys..

[58] Kumar R., Fredrickson G. H. (2009). Theory of polyzwitterion conformations. J. Chem. Phys..

[59] Doi, M. ; Edwards, S. F. The Theory of Polymer Dynamics; Oxford University Press: New York, 1986.

[60] Rumyantsev A. M., Gavrilov A. A., Johner A. (2024). Complete Diagram of
Conformational
Regimes for Polyampholytic Disordered Proteins. Macromolecules.

[61] Ghosh S., Kundagrami A. (2024). Effect of counterion size on polyelectrolyte
conformations
and thermodynamics. J. Chem. Phys..

[62] Glisman A., Mantha S., Yu D., Wasserman E. P., Backer S., Wang Z.-G. (2024). Multivalent Ion-Mediated Polyelectrolyte
Association and Structure. Macromolecules.

[63] Kundagrami A., Muthukumar M. (2010). Effective
Charge and Coil–Globule Transition
of a Polyelectrolyte Chain. Macromolecules.

[64] Kramarenko E. Y., Erukhimovich I. Y., Khokhlov A. R. (2002). The influence of ion pair formation
on the phase behavior of polyelectrolyte solutions. Macromol. Theory Simul..

[65] Solis F. J., Olvera de la Cruz M. (2000). Collapse of
flexible polyelectrolytes in multivalent
salt solutions. J. Chem. Phys..

[66] Brilliantov N. V., Kuznetsov D. V., Klein R. (1998). Chain Collapse and Counterion Condensation
in Dilute Polyelectrolyte Solutions. Phys. Rev.
Lett..

[67] Ha B.-Y., Thirumalai D. (1992). Conformations
of a polyelectrolyte chain. Phys. Rev. A.

[68] Zenga X., Ruffa K. M., Pappua R. V. (2022). Competing
interactions give rise
to two-state behavior and switch-like transitions in charge-rich intrinsically
disordered proteins. Proc. Natl. Acad. Sci.
U. S. A..

[69] Das R. K., Pappu R. V. (2013). Conformations of
intrinsically disordered proteins
are influenced by linear sequence distributions of oppositely charged
residues. Proc. Natl. Acad. Sci. U.S.A..

[70] Sawle L., Ghosh K. (2015). A theoretical method
to compute sequence dependent configurational
properties in charged polymers and proteins. J. Chem. Phys..

[71] Ha B.-Y., Thirumalai D. (1995). Electrostatic
Persistence Length of a Polyelectrolyte
Chain. Macromolecules.

[72] Cherstvy A. G. (2010). Collapse
of Highly Charged Polyelectrolytes Triggered by Attractive Dipole–Dipole
and Correlation-Induced Electrostatic Interactions. J. Phys. Chem. B.

[73] Jusufi A., Borisov O., Ballauff M. (2013). Structure formation in polyelectrolytes
induced by multivalent ions. Polymer.

[74] Katkar H. H., Muthukumar M. (2018). Role of non-equilibrium
conformations on driven polymer
translocation. J. Chem. Phys..

[75] Tesei G., Trolle A. I., Jonsson N., Betz J., Knudsen F. E., Pesce F., Johansson K. E., Lindorff-Larsen K. (2024). Conformational
ensembles of the human intrinsically disordered proteome. Nature.

[76] Muthukumar M. (2024). Dipole Theory
of Polyzwitterion Microgels and Gels. Gels.

[77] Rubinstein, M. ; Colby, R. H. Polymer Physics; Oxford University Press: New York, 2003.

[78] Wittmer J., Johner A., Joanny J. F. (1993). Random and Alternating Polyampholytes. Europhys. Lett..

[79] Ueda T., Oshida H., Kurita K., Ishihara K., Nakabayashi N. (1992). Preparation
of 2-Methacryloyloxyethyl Phosphorylcholine Copolymers with Alkyl
Methacrylates and Their Blood Compatibility. Polym. J..

[80] Muthukumar, M. Physics of Charged Macromolecules-Synthetic and Biological Systems; Cambridge University Press: New York, 2023.

[81] Rumyantsev A. M., Johner A. (2023). Salt-Added
Solutions of Markov Polyampholytes: Diagram
of States, Antipolyelectrolyte Effect, and Self-Coacervate Dynamics. Macromolecules.

[82] Moreno A. J., Lo Verso F., Arbe A., Pomposo J. A., Colmenero J. (2016). Concentrated
Solutions of Single-Chain Nanoparticles: A Simple Model for Intrinsically
Disordered Proteins under Crowding Conditions. J. Phys. Chem. Lett..

[83] de
Molina P. M., Le T. P., Iturrospe A., Gasser U., Arbe A., Colmenero J., Pomposo J. A. (2022). Neat Protein Single-Chain Nanoparticles from Partially
Denatured BSA. ACS Omega.

[84] Jiang X., Feng Q., Yang Y., Ge L., Cui Y., Zhao M., Jiang B. (2025). Polypeptide-Folded Artificial Ferroprotein
Promotes Ferroptosis in Multiple Tumor Cells. Biomacromolecules.

[85] Dobrynin A. V., Rubinstein M., Obukhov S. P. (1996). Cascade of Transitions
of Polyelectrolytes
in Poor Solvents. Macromolecules.

[86] Khokhlov A. R. (1980). On the
collapse of weakly charged polyelectrolytes. J. Phys. A: Math. Gen..

[87] Kantor Y., Kandar M. (1994). Excess Charge in Polyampholytes. Europhys. Lett..

[88] Solis F. J., de la Cruz M. O. (1998). Variational
Approach to Necklace Formation in Polyelectrolytes. Macromolecules.

[89] Jeon J., Dobrynin A. V. (2007). Necklace Globule
and Counterion Condensation. Macromolecules.

[90] Tang H., Liao Q., Zhang P. (2014). Conformation
of polyelectrolytes
in poor solvents: Variational approach and quantitative comparison
with scaling predictions. J. Chem. Phys..

[91] Everaers R., Milchev A., Yamakov V. (2002). The electrostatic persistence length
of polymers beyond the OSF limit. Eur. Phys.
J. E.

[92] Ullner M. (2003). Comments on
the Scaling Behavior of Flexible Polyelectrolytes within the Debye-Hückel
Approximation. J. Phys. Chem. B.

[93] Murnen H. K., Rosales A. M., Dobrynin A. V., Zuckermann R. N., Segalman R. A. (2013). Persistence length of polyelectrolytes
with precisely
located charges. Soft Matter.

[94] Ghosh S., Li X., Reed C. E., Reed W. F. (1990). Apparent persistence lengths and
diffusion behavior of high molecular weight hyaluronate. Biopolymers.

[95] Lopez C. G., Matsumoto A., Shen A. Q. (2024). Dilute polyelectrolyte solutions:
recent progress and open questions. Soft Matter.

[96] Tricot M. (1984). Comparison
of experimental and theoretical persistence length of some polyelectrolytes
at various ionic strengths. Macromolecules.

[97] Barrat J. L., Joanny J. F. (1993). Barrat. Europhys. Lett..

[98] Odijk T. (1979). Possible scaling
relations for semidilute polyelectrolyte solutions. Macromolecules.

[99] Odijk T., Houwaart A. C. (1978). Theory of excluded-volume effect of a polyelectrolyte
in a 1–1 electrolyte solution. J. Polym.
Sci., Polym. Lett. Ed..

[100] Odijk T., Mandel M. (1978). Influence of chain-flexiblity on
colligative properties of polyelectrolyte solutions. Physica A.

[101] Skolnick J., Fixman M. (1977). Electrostatic persistence length
of a wormlike polyelectrolyte. Macromolecules.

[102] Zhang L., Zhang X. Z., Lyu J. T., Yu L. X. Z., Wang C. Y., Sun Z. Y., Lu Z. Y., Qian H. J. (2024). Surface-Cross-linked
Protein-like Single-Chain Nanoparticle Globules Unexpectedly Stabilized
with a Low Cross-linking Degree. Macromolecules.

[103] Li L., Hou Z. (2023). Crosslink-Induced Conformation
Change of Intrinsically
Disordered Proteins Have a Nontrivial Effect on Phase Separation Dynamics
and Thermodynamics. J. Phys. Chem. B.

[104] Robles-Hernández B., de Molina P. M., Asenjo-Sanz I., Gonzalez-Burgos M., Pasini S., Pomposo J. A., Arbe A., Colmenero J. (2024). Dynamics of Single-Chain Nanoparticles
under Crowding: A Neutron Spin Echo Study. Macromolecules.

[105] Avecilla A. R. C., Thomas J., Quiroz F. G. (2025). Genetically-Encoded
Phase Separation Sensors Enable High-Fidelity Live-Cell Probing of
Biomolecular Condensates. ACS Sens..

