# Supplementary material for: Size, Conformation, and Local Domains of Single-Chain Nanoparticles with Intrachain Covalent, Dipolar, and Electrostatic Interactions: Toward Artificial Intrinsically Disordered Proteins
Source: Macromolecules. 2025 Jul 24;58(15):8536–54. doi: 10.1021/acs.macromol.5c01317 (PMC12356082; doi:10.1021/acs.macromol.5c01317)
Supplement: Supplementary file 1 [file ma5c01317_si_001.pdf]

Supporting Information

Size, Conformation and Local Domains of Single-Chain Nanoparticles with Intrachain Covalent, Dipolar and Electrostatic Interactions: Toward Artificial Intrinsically Disordered Proteins

Mikel Iguaran, Sara Gutierrez-Lkourt, Ester Verde-Sesto, Armando Maestro and José A. Pomposo*

* E-mail: Josetxo.pomposo@ehu.eus

Semi-Diluted Solutions of Single-Chain Nanoparticles with Intrachain Covalent and Dipolar Interactions

The critical concentration, $c*$, that separates the diluted ($c \leq c*$) and semi-diluted ($c > c*$) regimes is given by:[1]

$$c^* \approx \frac{N}{R_{1d}^3}, \qquad \text{(S1)}$$

where R_{1d} is given in Table 3 of the main text for $|s^2 v_d| \ll b^3$, $|s^2 v_d| = b^3$, and $|s^2 v_d| \gg b^3$, respectively. For $c > c*$ we can distinguish three cases depending on the balance between attractive dipolar and repulsive excluded volume interactions:

- $|s^2 v_d| \ll b^3$: According to scaling arguments and the ESN model, the SCNPs will adopt an ideal conformation above c^* such as $R_{c1d} \propto N^{3/8}$.
- $|s^2 v_d| = b^3$: The SCNPs will retain the ideal conformation above c^* such as $R_{c1d} \propto N^{3/8}$.
- $|s^2 v_d| \gg b^3$: The SCNPs will retain the globule conformation above c^* such as $R_{c1d} \propto N^{1/3}$.

Semi-Diluted Solutions of a Linear Precursor of Single-Chain Nanoparticles with Intrachain Covalent, Dipolar and Electrostatic Interactions

The critical concentration, $c*$, that separates the diluted ($c \leq c*$) and semi-diluted ($c > c*$) regimes is now:[1]

$$c^* \approx \frac{N}{(R_{0de})^3}, \qquad \text{(S2)}$$

where R_{0de} is given by eq. 32, 38 and 41 of the main text for $|s^2 v_d| \ll b^3$, $|s^2 v_d| = b^3$, and $|s^2 v_d| \gg b^3$, respectively. For $c > c*$, as in the case of semi-dilute solutions of neutral chains,[1] it is the correlation length ξ that determines the solution properties. From the classical scaling theory of PE solutions,[2] the dependence of the correlation length, ξ, and precursor size, R_{c0de}, on concentration, c, will be given by:

$$\xi \approx R_{0de} \left(\frac{c}{c*}\right)^{-\frac{1}{2}}, \qquad \text{(S3)}$$

and

$$R_{c0de} \approx R_{0de}\left(\frac{c}{c*}\right)^{-\frac{1}{4}} \propto N^{1/2}\,, \qquad \text{(S4)}$$

so the expected conformation of the precursor in the semidilute regime is that of a random walk (ν = ½). Regarding the local domain size, both D_e and g_e do not depend on c, so the expressions given in Table 2 for the cases: $|s^2 v_d| \ll b^3$, $|s^2 v_d| = b^3$, and $|s^2 v_d| \gg b^3$ remain valid also for the semi-dilute regime. The semi-dilute regime is expected to extend from c ~ c* until a concentration c** at which ξ = D_e (*i.e.,* until complete screening of electrostatic interactions)[2] such as:

$$c^{**} = c^{*}\left(\frac{R_{0\mathrm{de}}}{D_e}\right)^{2}. \qquad \text{(S5)}$$

Semi-Diluted Solutions of Single-Chain Nanoparticles with Intrachain Covalent, Dipolar and Electrostatic Interactions

The critical concentration, c*, that separates the diluted ($c \le c$*) and semi-diluted ($c > c$*) regimes is now:[1]

$$c^{*} \approx \frac{N}{(R_{1de})^3}\,, \qquad \text{(S6)}$$

where R_{1de} is given by eq. 53, 58 and 41 of the main text for $|s^2 v_d| \ll b^3$, $|s^2 v_d| = b^3$, and $|s^2 v_d| \gg b^3$, respectively. For c > c*, the dependence of the correlation length, ξ, and SCNP size, R_c, on concentration, c, are given by:

$$\xi \approx R_{1de}\left(\frac{c}{c*}\right)^{-\frac{1}{2}}\,, \qquad \text{(S7)}$$

and

$$R_{c1de} \approx R_{1de}\left(\frac{c}{c*}\right)^{-\frac{1}{4}} \propto N^{1/2}\,, \qquad \text{(S8)}$$

so the expected conformation of the SCNPs in the semidilute regime is that of a random walk (ν = ½). Regarding the local domain size, both D_c and g_c do not depend on c, so the expressions given in Table 4 for the cases: $|s^2 v_d| \ll b^3$, $|s^2 v_d| = b^3$, and $|s^2 v_d| \gg b^3$ remain valid also for the semi-dilute regime. The semi-dilute regime extends from c ~ c* until a concentration c** at which ξ = D_c (*i.e.,* until complete screening of electrostatic interactions)[2] such as:

$$c^{**} = c^{*}\left(\frac{R_{1\mathrm{de}}}{D_c}\right)^2. \qquad \text{(S9)}$$

Collapsed Conformations of SCNPs prepared at Extremely Low Intrachain Crosslinking Degree

Very recently, Qian and coworkers[3] reported a flow photochemistry method to obtain collapsed conformations of SCNPs at extremely low intrachain crosslinking degree. In particular, these authors selected random copolymers of styrene (S) and 4-acryloyloxybenzophenone (ABP) as SCNPs precursors and acetone as a poor solvent for S but a good solvent for ABP. The ABP monomer which was the reactive monomer for intrachain photo-crosslinking was used at very low amount in the precursors (from 1.6 to 5.7 mol%).

In particular, the hydrodynamic diameter of the precursor with 5.7 mol% of ABP (N = 224, x = 0.057) changed from 7.0 nm in toluene (good solvent for both S and ABP) to 5.6 nm in acetone (poor solvent for S). We can rationalize this size reduction within the model of SCNPs with intrachain covalent and dipolar interactions as if it was due to attractive dipolar interactions between S monomers. First, we estimate an "effective" monomer length of b = 0.27 nm from $b = R_0 / N^{3/5}$ (R_0 = 7.0 nm, N = 224). Next, from eq. 9 in the main text we estimate an "effective" value of v_{d0} = – 0.015 nm^3 by using s = 0.943, b = 0.27 nm and R_{d0} / R_0 = 0.8. Then, we can explore the ability of the model to predict the size of the precursors with 1.6 (N = 190), 2.2 (N = 199), 3.2 (N = 219) and 4.3 (N = 208) mol% of ABP. A comparison of the size of the different precursors as calculated from eq. 8 in the main text with the experimental data reported by Qian and coworkers[3] is shown in Figure S1. Also included in this Figure are the predictions of the model for the size of the corresponding SCNPs as calculated from eq. 18 by taking a value of A = 10 nm^{-2}.

Overall, the predictions compare very good agreement with the experimental results, as shown in Figure S1. Moreover, if we set v_{d0} = 0 nm^3 in the model, the predicted size of the SCNPs increases by a 25-30 % (red open triangles in Figure S1) so the model allows one to *visualize* the contribution of the attractive interactions to the real size of these SCNPs in a straightforward way.

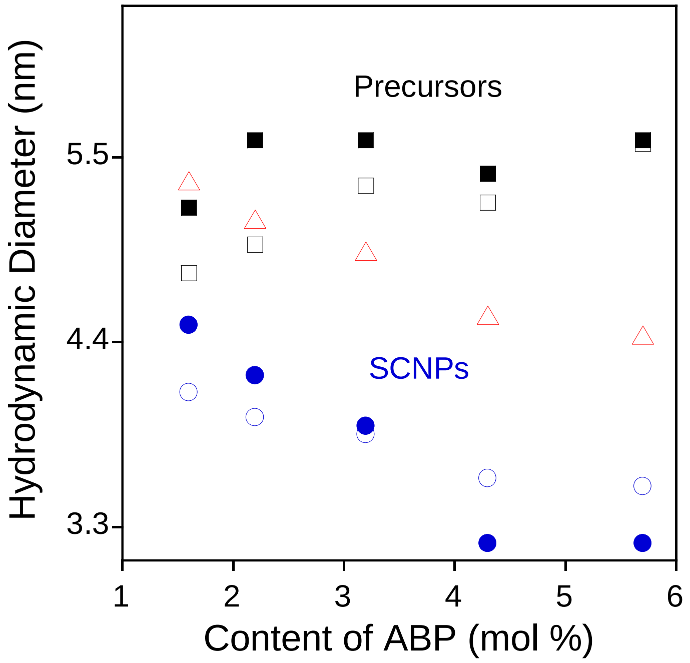

Figure S1. Comparison of model predictions (open symbols) and experimental results (closed symbols) for SCNPs synthesized at extremely low intrachain crosslinking degree.[3] Open squares (precursors) and open circles (SCNPs) are predictions with v_{d0} = – 0.015 nm^3. Open triangles (SCNPs) correspond to predictions with v_{d0} = 0 nm^3 (see text for details).

Scaling of the Size with the Intrachain Crosslinking Degree Observed in Coarse-Grained MD simulations of Globally Neutral IDPs

Very recently, Li and Hou[4] performed coarse-grained MD simulations of globally neutral IDPs in which intramolecular crosslinks were used as conformational regulators. These authors investigate conformation−phase separation relationships by applying a residue based coarse-grained model of IDPs, in which a fraction of preformed intrachain crosslinks was introduced to simulate proteins with a diversity of conformation profiles. To exclude linking-position effects, each chain in the simulation was endowed with a unique pattern of crosslink sites, reflecting an ensemble averaged system under a certain crosslink ratio in order to modulate the structural heterogeneity of IDPs. The results of these simulations validate the model of non-ionic SCNPs -as primitive mimics of IDPs- for which the predicted scaling of the size (R) on the intrachain crosslinking degree (x) is $R \propto x^{-1/5}$. Figure 2 illustrates the excellent agreement of model predictions and MD simulations results.

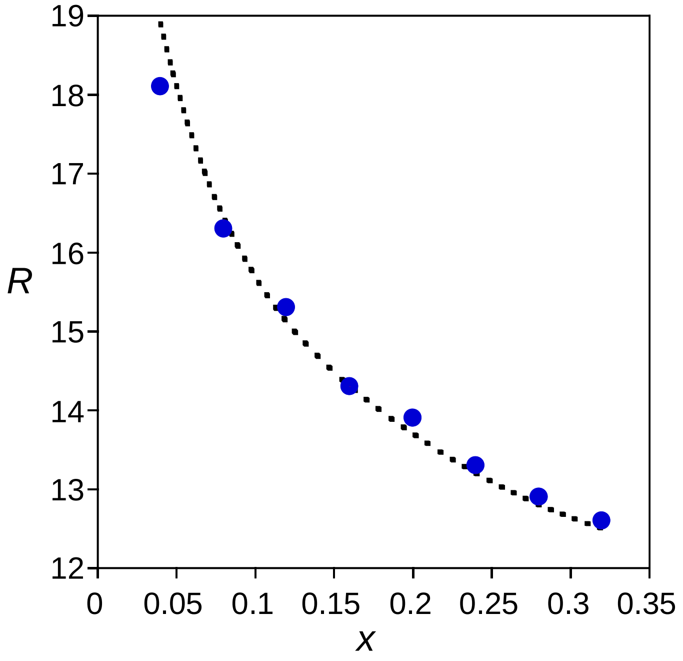

Figure S2. Comparison of model predictions about the scaling of the size (*R*) with the intrachain crosslinking degree (*x*) for non-ionic SCNPs (dotted line, corresponding to $R \propto x^{-1/5}$) and MD simulation results of globally neutral IDPs[4] (closed circles).

References

(1) Rubinstein, M.; Colby, R. H. *Polymer Physics*; Oxford University Press: New York, 2003.

(2) Dobrynin, A. V.; Colby, R. H.; Rubinstein, M. Scaling Theory of Polyelectrolyte Solutions. *Macromolecules* **1995**, *28*, 1859-1871.

(3) Zhang, L.; Zhang, X. Z.; Lyu, J. T. ; Yu, L. X. Z.; Wang, C. Y.; Sun, Z. Y.; Lu, Z. Y.; Qian, H. J. Surface-Cross-linked Protein-like Single-Chain Nanoparticle Globules Unexpectedly Stabilized with a Low Cross-linking Degree. *Macromolecules* **2024**, 57, 858-868.

(4) Li, L.; Hou, Z. Crosslink-Induced Conformation Change of Intrinsically Disordered Proteins Have a Nontrivial Effect on Phase Separation Dynamics and Thermodynamics. *J. Phys. Chem. B* **2023**, *127*, 5018-5026.
